# Impact of Imaging Biomarkers and AI on Breast Cancer Management: A Brief Review

**DOI:** 10.3390/cancers15215216

**Published:** 2023-10-30

**Authors:** Gehad A. Saleh, Nihal M. Batouty, Abdelrahman Gamal, Ahmed Elnakib, Omar Hamdy, Ahmed Sharafeldeen, Ali Mahmoud, Mohammed Ghazal, Jawad Yousaf, Marah Alhalabi, Amal AbouEleneen, Ahmed Elsaid Tolba, Samir Elmougy, Sohail Contractor, Ayman El-Baz

**Affiliations:** 1 Diagnostic and Interventional Radiology Department, Faculty of Medicine, Mansoura University, Mansoura 35516, Egypt; gehadsaleh@mans.edu.eg (G.A.S.);; 2 Computer Science Department, Faculty of Computers and Information, Mansoura University, Mansoura 35516, Egyptastolba@mans.edu.eg (A.E.T.);; 3 Electrical and Computer Engineering Department, School of Engineering, Penn State Erie, The Behrend College, Erie, PA 16563, USA; ave5429@psu.edu; 4 Surgical Oncology Department, Oncology Centre, Mansoura University, Mansoura 35516, Egypt; omarhamdy@mans.edu.eg; 5 Bioengineering Department, University of Louisville, Louisville, KY 40292, USA; 6 Electrical, Computer, and Biomedical Engineering Department, Abu Dhabi University, Abu Dhabi 59911, United Arab Emirates; mohammed.ghazal@adu.ac.ae (M.G.);; 7 The Higher Institute of Engineering and Automotive Technology and Energy, New Heliopolis, Cairo 11829, Egypt; 8 Department of Radiology, University of Louisville, Louisville, KY 40202, USA

**Keywords:** breast cancer, BI-RADS, molecular imaging, biomarkers, PET-CT

## Abstract

**Simple Summary:**

Artificial intelligence (AI) has seamlessly integrated into the medical field, especially in diagnostic imaging, thanks to ongoing AI advancements. It is widely used in various medical applications. In the context of breast cancer (BC), machine learning and deep learning are extensively employed for automating diagnosis, segmenting relevant data, and predicting pre-treatment tumor response to new adjuvant chemotherapy (NAC). Recent research has shown promising results with deep learning algorithms in BC diagnosis, accurately identifying specific features, demonstrating AI’s potential to enhance BC diagnosis and analysis precision and efficiency. Additionally, utilizing non-ionized modalities, apart from ionized mammograms, has a substantial impact on the diagnosis process.

**Abstract:**

Breast cancer stands out as the most frequently identified malignancy, ranking as the fifth leading cause of global cancer-related deaths. The American College of Radiology (ACR) introduced the Breast Imaging Reporting and Data System (BI-RADS) as a standard terminology facilitating communication between radiologists and clinicians; however, an update is now imperative to encompass the latest imaging modalities developed subsequent to the 5th edition of BI-RADS. Within this review article, we provide a concise history of BI-RADS, delve into advanced mammography techniques, ultrasonography (US), magnetic resonance imaging (MRI), PET/CT images, and microwave breast imaging, and subsequently furnish comprehensive, updated insights into Molecular Breast Imaging (MBI), diagnostic imaging biomarkers, and the assessment of treatment responses. This endeavor aims to enhance radiologists’ proficiency in catering to the personalized needs of breast cancer patients. Lastly, we explore the augmented benefits of artificial intelligence (AI), machine learning (ML), and deep learning (DL) applications in segmenting, detecting, and diagnosing breast cancer, as well as the early prediction of the response of tumors to neoadjuvant chemotherapy (NAC). By assimilating state-of-the-art computer algorithms capable of deciphering intricate imaging data and aiding radiologists in rendering precise and effective diagnoses, AI has profoundly revolutionized the landscape of breast cancer radiology. Its vast potential holds the promise of bolstering radiologists’ capabilities and ameliorating patient outcomes in the realm of breast cancer management.

## 1. Introduction

Breast cancer (BC) stands as a significant global health concern, emerging as the most commonly diagnosed cancer and holding the fifth rank among the leading causes of cancer-related deaths worldwide [1]. Diverse risk factors contribute to the variable likelihood of BC, encompassing factors such as advanced age, tobacco exposure, obesity, usage of hormonal therapy or oral contraceptives, breast density, genetic mutations, and familial history of BC [2]. Predominantly, genetic mutations linked to breast cancer involve the BRCA1/2 genes.

Imaging plays a vital role in detecting, staging, assessing response, and detecting early recurrence in patients with BC [3]. The use of standardized terminology and structured reporting allows radiologists to convey their findings clearly and succinctly in breast imaging to referring physicians [4]. A critical foundation in this context is the Breast Imaging Reporting and Data System (BI-RADS), developed by the American College of Radiology (ACR). However, an updated and comprehensive strategy is necessary to incorporate these cutting-edge techniques, as new imaging modalities have emerged since the publication of the 5th edition of BI-RADS.

In this review article, we delve into a concise narrative alongside updates on the Breast Imaging Reporting and Data System (BI-RADS) lexicon, exploring advanced mammography techniques, ultrasonography (US), and magnetic resonance imaging (MRI). The discussion encompasses a range of recent and valuable imaging modalities that have gained prominence subsequent to the latest BI-RADS 5th edition, significantly enhancing breast cancer (BC) diagnosis. This progress underscores the need for their seamless integration in forthcoming BI-RADS updates. This review further presents current insights into BC molecular subtypes, molecular breast imaging (MBI), and diagnostic imaging biomarkers. The subsequent focus shifts to diverse treatment strategies and the precise evaluation of treatment responses. Alongside these imaging advancements, the revolutionary potential of artificial intelligence (AI) in breast cancer detection and treatment comes to the fore. The remarkable capacity of AI algorithms to analyze extensive image datasets, discerning subtle patterns imperceptible to the human eye, holds transformative promise. Radiologists stand to augment precision, efficiency, and overall diagnostic capabilities in breast cancer through AI utilization. This comprehensive review accentuates the supplementary value brought forth by AI applications in BC diagnosis and management.

## 2. Search Methodology

This study provides a concise overview of BI-RADS history, advanced mammography techniques, ultrasonography (US), magnetic resonance imaging (MRI), diagnostic imaging biomarkers, the assessment of treatment response, and various AI-based methods for breast cancer diagnosis and detection. The methodology involved a comprehensive search strategy that aimed to identify relevant studies, reports, and resources pertinent to breast cancer. The number of papers focusing on breast cancer imaging has experienced a significant increase. In this context, our concentration lies in the sphere of recent breast cancer research conducted within the past decade. A manual examination was conducted on English-language articles and research papers published prior to July 2023, utilizing electronic databases such as Embase, WebofScience, PubMed, and Google Scholar. The search encompassed terms such as “breast cancer” and “BI-RADS”, as well as “detection”, “diagnosis”, and “management”, combined with either “machine learning”, “ML”, “artificial intelligence”, “AI”, “deep learning”, or “DL”. A prerequisite for inclusion was the status of being an original research article either published, accepted for publication, or available online in English. Studies involving considerations of sex or age were incorporated.

## 3. BI-RADS Overview

In this section, we will provide an overview of the various versions of BI-RADS, the assessment categories and recommendations within BI-RADS, as well as introduce the BI-RADS lexicon.

### 3.1. Updates of BI-RADS

To delineate breast imaging findings and outcomes, radiologists employ a standardized system known as BI-RADS. Initially introduced by the American College of Radiology (ACR) in 1992 to address inconsistencies in mammography reporting, subsequent editions of BI-RADS were published in 1995, 1998, and 2003 [5]. The current 5th edition of BI-RADS was released in February 2014 and was updated through collaboration between breast imaging radiologists and specialized clinicians focused on breast health [6]. It has evolved from a simple mammography lexicon to a comprehensive lexicon encompassing mammography, ultrasound (US), and magnetic resonance imaging (MRI). Lexical descriptors were formulated to account for both benign and malignant lesions, eliminating ambiguity and enhancing communication with referring clinicians [7]. Structured BI-RADS reports provide assessment categories, encompassing breast density, description of findings, and management recommendations [8,9].

### 3.2. BI-RADS Assessment Categories and Recommendations

The assessment categories provide estimates of the likelihood of malignancy in breast lesions and offer management recommendations for each category. Although the BI-RADS categories and recommendations remain unchanged in the 5th edition, they are now decoupled and can exist independently. This modification allows for flexibility in management recommendations while retaining precise medical assessments based on imaging findings [8]. Categories 0, 1, and 2 are applied during screening mammography, ultrasound, and MRI, yielding similar consequences. Categories 3, 4, and 5 are assigned after a comprehensive diagnostic imaging assessment (Table 1).

Mammographic lesions properly corroborated as BI-RADS category 3 involve a solitary circumscribed mass, a focal asymmetry without corresponding sonographic correlate, and a punctate grouped calcification [10,11]. A recent study revealed that category 3 assignment after recall from screening mammography is applicable, with a 1.86% accumulative cancer yield over a 2-year follow-up, verifying the need for short-interval follow-up of BI-RADS category 3 findings [12]. Distinctive US criteria for BI-RADS category 3 comprise nonpalpable circumscribed oval masses implying fibroadenoma and complicated cysts [8]. Numerous studies reported that the short-term follow-up offered by BI-RADS category 3 granted early-stage breast cancer detection, with no harmful consequences credited to a short delay in diagnosis [8,13,14].

BI-RADS category 4 has a wide range of anticipated risk of malignancy and accordingly it has been subdivided into three categories in both US and mammography to overcome radiology–pathology discordance [15]. Category 4A can be utilized to address cases that may be securely downgraded, by using potential supplemental technologies, such as contrast-enhanced mammography (CEM) [16]. Evidence for the MRI subdivision of category 4 is still limited [8].

BI-RADS category 5 indicates a very high probability of malignancy and is utilized identically through mammography, US, and MRI. Careful radiology–pathology correlation is essential if percutaneous biopsy of a category 5 lesion shows a benign histopathology, to establish if repeat image-guided biopsy or surgical biopsy is the ideal following step [14]. BI-RADS category 6 was announced in the 4th edition [8]

### 3.3. BI-RADS Lexicon

A lexicon serves as a collection of consistent terms used to concisely and accurately describe imaging findings. Various studies have certified lexicons for different imaging modalities [16,17]. The 5th edition of BI-RADS harmonizes terminology across mammography, ultrasound (US), and breast MRI, enabling precise correspondence between these modalities, facilitating communication among radiologists, and ensuring consistent reporting to referring clinicians [8]. However, lexicon descriptors developed for specific techniques may not always be adaptable for harmonization, especially in the case of the MR dynamic contrast enhancement (DCE) technique and kinetic assessment of signal intensity changes. As a result, the MRI lexicon includes unique descriptors designed specifically for quantifying contrast kinetics [18].

Several breast imaging modalities that have emerged since the publication of the BI-RADS 5th edition lack a distinct lexicon. These modalities include contrast-enhanced mammography (CEM), positron emission mammography (PEM), and molecular breast imaging (MBI). Studies have attempted to adapt existing descriptors for these recent modalities. For instance, various studies assessing the applicability of MRI BI-RADS descriptors to CEM have indicated that mass lesion features yield the most consistent results across MRI and CEM [16,17]. Similarly, the BI-RADS lexicon has been customized for MBI and PEM, incorporating recommendations for lesion features and relevant molecular agent uptake [19,20]. Moreover, in the future, contrast kinetics lexicon descriptors may be employed to advance technologies such as CEM. Perry et al. [21] used the existing descriptors for CEM by employing mammographic lesion descriptors for low-energy images and MRI descriptors (BPE and enhancement characteristics) for recombined images.

## 4. BI-RADS Supported Modalities: Findings and Updated Techniques

As illustrated in Section 3.3, the 5th edition of BI-RADS supports three modalities: mammography, ultrasound, and MRI. In this section, we will outline the distinct findings within BI-RADS for each of these supported modalities, while also providing an overview of the updates pertaining to breast cancer in each modality.

### 4.1. BI-RADS Mammography Findings

Density in mammography was previously categorized as 1 through 4, leading to confusion with assessment categories. In the BI-RADS 5th edition, breast composition is now assessed visually and classified as density levels a, b, c, or d, ranging from fatty to highly dense [8]. Breast density is determined by the presence of focal dense breast tissue that can obscure the detection of cancer, even in cases where the overall breast is non-dense. Higher breast density has been associated with an increased likelihood of findings being hidden by normal dense breast tissue on mammograms, a phenomenon referred to as the masking effect [22,23]. Architectural distortion and calcifications can serve as primary descriptors or associated features. Additionally, findings such as skin lesions, intramammary lymph nodes (LN), and solitary dilated ducts should be documented [8]. Mammography offers a significant advantage over other methods because it can detect microcalcifications, which are the initial manifestation in approximately 30–50% of nonpalpable breast cancers, particularly ductal carcinoma in situ (DCIS) [24]. A list of BI-RADS 5th edition Mammography findings can be found in Table 2.

### 4.2. Advancements in Mammographic Imaging Techniques

**Figure 1:**
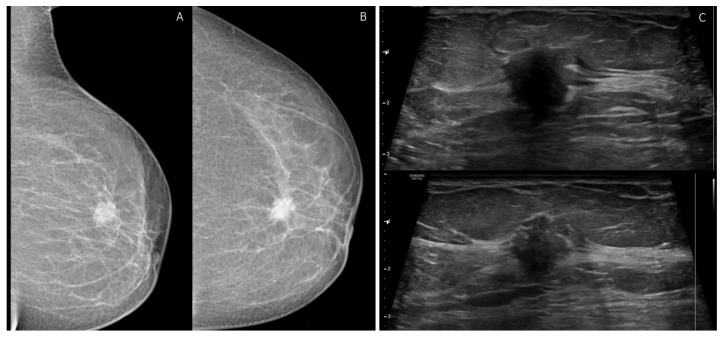
BI-RADS category 4C: 40-year-old woman with invasive ductal carcinoma. (**A**,**B**) left mediolateral oblique and craniocaudal digital mammogram show irregular high-density mass with spiculated margins. (**C**) transverse ultrasound shows an irregular mass with angular margins and posterior shadowing corresponding to mammographic findings. No suspicious axillary LNs.

Digital Mammography (DM) is the preferred method for breast cancer screening and early detection; however, it exhibits low sensitivity in dense breasts, potentially concealing underlying masses [25,26] (Figure 1). To address this limitation, new mammographic modalities have been introduced, namely digital breast tomosynthesis (DBT) and contrast-enhanced mammography (CEM). In the following section, we will provide an overview of each of these advancements.

**Digital Breast Tomosynthesis (DBT)** is an advancement in digital mammography (DM) that allows for the acquisition of three-dimensional (3D) volumetric data of thin breast sections [27]. DBT reduces the tissue overlap between dense breast parenchyma and underlying masses, enabling better visualization and accurate diagnosis of breast cancer cases that may be missed by DM [28]. Furthermore, DBT demonstrates enhanced diagnostic efficacy in evaluating dense breast tissue, particularly for initial BI-RADS 0 results and the identification of indeterminate BI-RADS 3 and 4 lesions, surpassing the capabilities of DM [29,30]. The combination of DBT and DM significantly enhances the performance of BI-RADS in diagnosing indeterminate breast lesions, and in some cases, DBT alone can lead to a change in the final BI-RADS classification [31].

**Contrast-Enhanced Mammography (CEM)** is a clinically available technique, performed in a modified mammography unit, with a similar examination time as DM [32]. CEM improves breast cancer (BC) detection by visualizing malignant enhancement after the injection of an intravenous contrast agent [33]. Multiple previous studies have concluded that CEM and Contrast-enhanced MRI have equivalent diagnostic performance for detecting BC, with CEM offering the advantages of reduced cost and examination time [32,33,34,35,36]. Recent studies have also recommended CEM for high-risk and dense breast screening [35]. To study and report enhancing lesions found on CEM, a standardized lexicon of morphological descriptors is required. A previous study reported that the MRI BI-RADS lexicon of morphology descriptors can confidently be applied to illustrate lesions on CEM [37]. A recently published meta-analysis showed that CE-MRI revealed higher sensitivity and negative likelihood ratios to exclude malignancy compared to CEM [33].

### 4.3. BI-RADS Ultrasound Findings

US is regarded as a valuable complement to mammography and MRI [38]. The lexicon for breast ultrasound has been revised to improve consistency across all imaging modalities, streamline reporting processes, and incorporate advanced techniques like elastography. Furthermore, the “special cases” category has been expanded in the BI-RADS 5th edition [7]. Detailed ultrasonic findings in accordance with the BI-RADS 5th edition can be found in Table 3.

### 4.4. Advancements in US Techniques

Since only a small percentage of BI-RADS-US 3 lesions (3%) were determined to be malignant, causing delays in cancer diagnosis, and a large portion of BI-RADS-US 4A lesions were found to be benign, leading to excessive and unnecessary biopsies, it is necessary to investigate an appropriate diagnostic predictor of malignancy in BI-RADS-US 3 and BI-RADS-US 4A lesions [39]. To overcome these limitations, updated US techniques have been investigated, including US elastography and contrast-enhanced ultrasound (CEUS), as illustrated below.

**US-Elastography** is a noninvasive ultrasound technique utilized to generate images that depict the relative hardness or stiffness of tissue, as cancerous tissue typically exhibits greater firmness compared to the surrounding normal breast parenchyma [40]. Shear wave elastography (SWE) and ultrasound strain elastography (USE) play crucial roles in continuously improving elastography techniques. A recent prospective study [41] has demonstrated that combining B-mode ultrasound with both USE and SWE results in an enhanced BI-RADS category assessment, with USE achieving statistically significantly higher accuracy than SWE. USE is generated through manual pressure and release, allowing for real-time calculation of tissue elasticity [42]. The five-point elasticity score is determined by evaluating the level and pattern of strain observed within the lesion and its surrounding tissue, as illustrated in Table 4 [43]. Furthermore, the integration of elastography into the BI-RADS system holds the potential to increase its diagnostic value, particularly in cases involving lesions smaller than 2 cm, as USE enhances the ability to differentiate between malignant and benign small lesions [44].

**Contrast-enhanced ultrasound (CEUS)** demonstrates favorable sensitivity and specificity when categorizing breast lesions, as it effectively delineates the microvascular architecture of these lesions [45,46]. In terms of enhancing breast lesions, CEUS images can be graded using a five-score system, as illustrated in Table 4 [47,48]. A previous study compared the diagnostic performance of conventional ultrasound (US), ultrasound elastography (USE), and CEUS in characterizing subcentimeter breast lesions, revealing that the combination of BI-RADS-US with CEUS yielded the highest diagnostic accuracy. Moreover, both USE and CEUS serve as viable alternatives to a biopsy for such small lesions [47].

**Table 4:** Scores for updated ultrasound techniques (USE and CEUS scores). USE: ultrasound strain elastography [43], CEUS: contrast-enhanced US [47].

Score	USE Score	CEUS Score
1	The entire lesion is uniformly colored in green	Ring-like enhancement, no internal enhancement.
2	The lesion is shadowed in green with focal blue spots	Iso- and synchronous enhancement of the lesion with the surrounding tissue. No clear outline.
3	The half of the lesion is green and half blue	Earlier enhancement of the lesion than neighboring tissue either heterogeneous or homogeneous. Clear margin. The lesion size is nearly equal to that demonstrated in a 2D image. Regular shape.
4	The whole lesion is blue or predominantly blue with a minimum green	Earlier enhancement of the lesion than neighboring tissue, typically heterogeneous. The lesion size is larger than that in the 2D image, the lesion still reveals a clear margin with or without a perfusion defect inside the lesions, no crab claw-like enhancement. Irregular shape.
5	The whole lesion and its neighboring area ware blue or blue with focal green spots	Heterogeneous enhancement of the lesion with a larger size than that in the 2D image. With or without perfusion defect. Crab claw-like enhancement with an unclear margin.

### 4.5. BI-RADS MRI Findings

**Figure 2:**
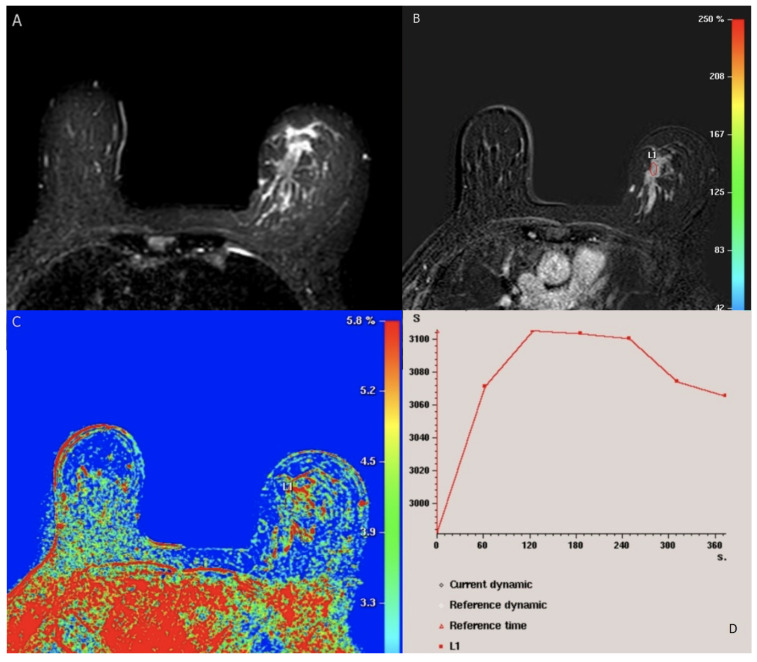
BI-RADS category 5: 49-year-old woman with Luminal (**A**) (positive ER and PR, negative Her2) lobular carcinoma. Axial fat-saturated T2-weighted MRI shows left breast hyperintense irregular mass. (**B**) axial T1-weighted fat-saturated dynamic contrast-enhanced MR image shows heterogenous segmental nonmass enhancement of the lesion (L1). (**C**) kinetic color overlay shows predominant yellow and red colors in the mass indicating plateau kinetics and washout respectively. (**D**) kinetic signal intensity time curve shows rapid initial phase and washout in the delayed phase. No suspicious axillary LNs.

Breast MRI plays a pivotal role in visualizing breast cancer. MRI boasts the highest sensitivity, ranging from 88% to 100% when compared to other breast imaging modalities (Figure 2) [49]. The BI-RADS lexicon has significantly improved the reliability of interpreting and reporting breast MR imaging [50]. It is important to note that background parenchymal enhancement (BPE) of breast tissue on MRI differs from mammography density or the MRI appearance of breast fibroglandular tissue. BPE has been established as an indicator of increased breast cancer risk, regardless of breast density [8]. The recommended timing for screening breast MRI was previously suggested during the second week of the menstrual cycle to minimize BPE [51,52]. However, recent research questions the validity of this suggestion, as menstrual cycle phases have shown no significant impact on reporting results [53]. Most contemporary MRI protocols are multiparametric [54,55], and detailed findings according to the 5th edition of BI-RADS MRI can be found in Table 5 [8].

### 4.6. Advancements in MRI Techniques

The various components of the multiparametric MRI protocol, which encompass the quantitative assessment of contrast medium enhancement and advanced diffusion-weighted MRI (DW-MRI) techniques, contribute significantly to enhancing the classification of lesions.

**Dynamic Contrast-Enhanced Magnetic Resonance Imaging (DCE-MRI)** assesses blood vessel permeability by using an intravenous contrast medium, gadolinium chelate, which shortens the local T1 time and results in a higher signal in T1-weighted images [56]. Neoangiogenesis in breast cancer leads to the formation of leaky vessels that facilitate faster extravasation of the contrast medium [57], resulting in rapid local contrast enhancement. Dynamic evaluation with time-signal intensity curves involves acquiring a series of T1-weighted images from five to seven minutes after contrast administration [58,59]. In malignant tumors with leaky vessels, the peak accumulation of the contrast medium has already occurred, and it is being washed out from the tumor. In benign lesions with vessels of lower permeability, the accumulation of the contrast medium within the vessel wall continues to show positive results, resulting in ongoing enhancement. This concept helps elucidate the significance of kinetic time-signal intensity curves. A persistent rise is most commonly seen in benign lesions, while a decrease in the late phase is typical of malignant tumors [60]. The curve suspicious for malignancy is often characterized by a “washout-plateau-persistent” pattern, observed in approximately 85% of malignant tumors [59,60]. Conversely, persistent time-signal intensity curves are rare in cancers, although they are a possibility in cases of ductal carcinoma in situ (DCIS) and more diffusely growing invasive tumors, especially lobular breast cancers [61].

**Diffusion-weighted magnetic resonance imaging (DW-MRI)** measures the random Brownian motion of water molecules within tissue, a motion influenced by tissue microstructure and cell density. Motion-sensitizing gradients (b factors) are applied to a T2-weighted echo-planar sequence [62,63]. Malignant tumors exhibit reduced water diffusion due to increased cell density, resulting in diffusion restriction and high DW-MRI signal intensity. To obtain high-quality DW-MRI scans, it is essential to select appropriate b values, minimize artifacts, ensure effective fat suppression, and maintain a satisfactory signal-to-noise ratio [63]. The apparent diffusion coefficient (ADC) serves as a numerical representation of diffusion values. Typically, ADC values are lower in malignant tumors, falling within the range of 0.8 to $1.3\times 10^{-3}$ mm ${}^{2}$/s, as opposed to benign lesions, where they usually range from 1.2 to $2.0\times 10^{-3}$ mm ${}^{2}$/s. This difference arises from the constrained diffusion properties of cancerous tissue [64]. When DW-MRI is conducted with *ab* value lower than 1000 s/mm ${}^{2}$, it demonstrates the highest precision in distinguishing between benign and malignant lesions [65,66].

**Figure 3:**
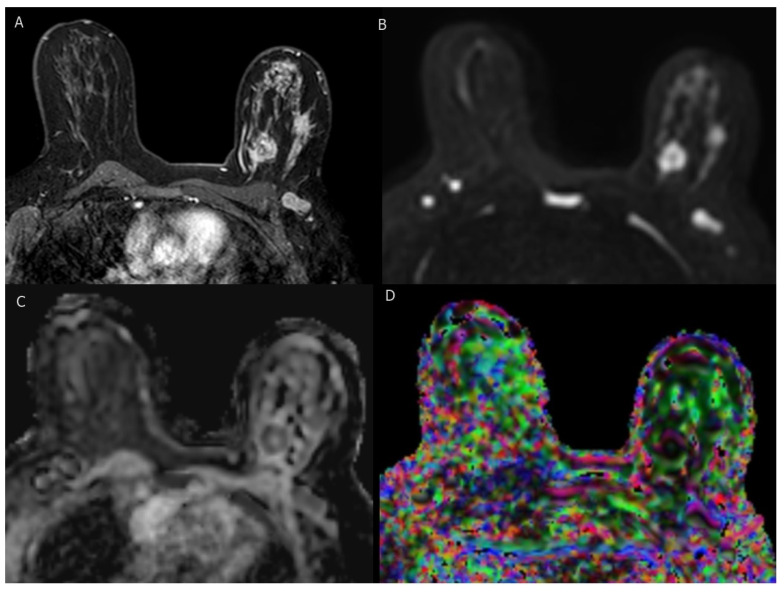
BI-RADS category 5: 37-year-old woman with triple-negative invasive ductal carcinoma. (**A**) axial T1-weighted fat-saturated dynamic contrast-enhanced MR image shows two irregular masses of heterogenous enhancement with ipsilateral suspicious axillary LN. (**B**–**D**) axial DWI (b = 1000), corresponding ADC map, and DTI image (colored FA map ) show marked diffusion restriction of two masses and axillary LN with low ADC values ($0.89\times 10^{-3}$ mm ${}^{2}$/s, $0.91\times 10^{-3}$ mm ${}^{2}$/s and $0.93\times 10^{-3}$ mm ${}^{2}$/s ) and high FA values (0.57, 0.56 and 0.54), respectively.

**Diffusion tensor magnetic resonance imaging (DTI-MRI)** not only measures the apparent diffusion coefficient (ADC) but also provides information about diffusion directionality [67]. Diffusion anisotropy arises from the alignment of water diffusion within the microstructure of breast tissue, which consists of ducts and lobules. DTI-MRI surpasses DW-MRI by enabling the investigation of water motion in six or more directions to fully characterize the diffusion tensor. Fractional anisotropy (FA) serves as a crucial metric for quantifying the extent of DTI directionality and has been a primary parameter in DTI studies [68]. More intricate three-dimensional diffusion patterns and their mean diffusivity are also considered. It has been postulated that malignant tumors may be linked to higher cell density and a more disorganized microstructure, resulting in decreased FA. However, the findings regarding DTI’s efficacy in differentiating between benign and malignant breast tumors are still a subject of debate. Some studies have reported a significant decrease in FA in malignant breast tumors compared to normal parenchyma, while others have found no significant differences [67] (see Figure 3).

## 5. Microwave Breast Imaging

The flaws of mammography, such as radiation exposure and uncomfortable breast compression, attract attention to research into alternate methods of imaging [69]. Microwave imaging (MWI) offers an emerging potential non-ionizing, non-compressive method for BC diagnosis [70]. MWI has been assigned as a potentially viable technique for identifying breast abnormalities [71], with reported high sensitivity for identifying cancer in denser breasts [72]. MWI uses electromagnetic radiation to deduce the dielectric characteristics within a set volume, named the imaging domain [69]. The Wavelia system is a first generation, low-power electromagnetic wave breast imaging tool, utilized by MVG Industries [73]. A recent study concluded that the Wavelia system demonstrated promising results in detecting benign and malignant breast lesions in a clinical setting [74]. SAFE (Scan and Find Early) is a novel MWI device envisioned for BC screening and early detection. A recent preliminary study compared SAFE results with US, mammography, and MRI, which revealed promising concordance with clinical reports, consequently encouraging additional SAFE clinical studies [75].

## 6. The Role of AI in the Detection and Diagnosis of Breast Cancer

By utilizing AI components, artificial intelligence (AI) aims to replicate human problem-solving and thought processes. A fundamental element of AI is machine learning (ML), which involves the utilization of image processing methods to extract characteristics or features from a given input dataset. Subsequently, the data are either graded through supervised learning or classified through unsupervised learning. In supervised learning, labeled input–output pairs are employed to classify data using classifiers such as support vector machines (SVM), random forests, and conventional neural networks. Deep learning (DL), a subset of machine learning, has gained popularity in the medical industry, with convolutional neural networks (CNNs) being the most frequently used deep learning networks. CNNs comprise multiple convolutional and fully connected layers to accomplish feature extraction and classification. In contrast, unsupervised learning classifies data based on patterns within the input data rather than labeled input–output pairs. AI has played a pivotal role in recent times, particularly in applications like early breast cancer (BC) detection and diagnosis. Various metrics are employed to address medical issues such as categorization, diagnosis, and early detection, enabling the assessment of the effectiveness of AI components. Below is a brief summary of these measures, with the total number of data samples denoted as TP + TN + FN + FP. The abbreviations represent true positive (TP), true negative (TN), false negative (FN), and false positive (FP), defined as follows:

- True negative (TN): both the classifier’s prediction and the test case were negative.
- True positive (TP): both the classifier’s prediction and the test case were negative.
- False negative (FN): the test cases yielded positive results, but the classifier’s prediction was negative
- False positive (FP): the test cases turned out to be negative, but the prediction was positive.

The following definitions in Table 6 apply to the performance measurements used in this study.

**Figure 4:**
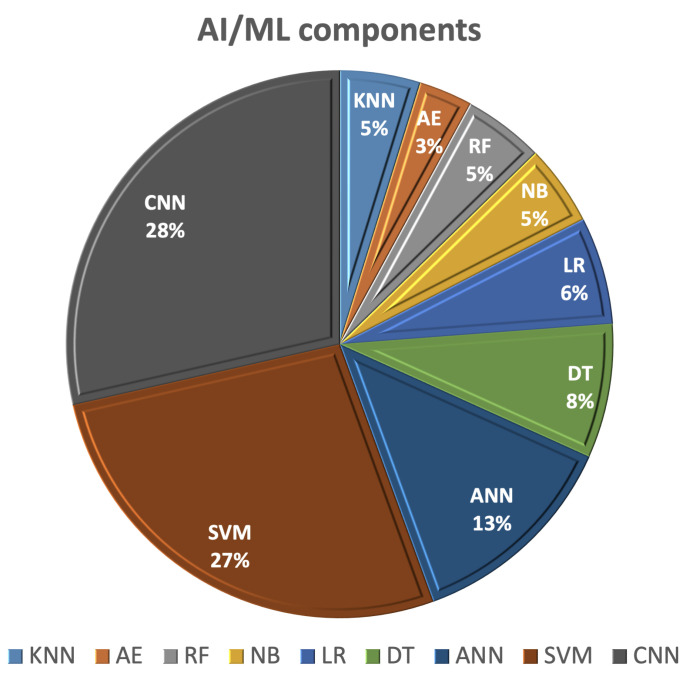
The most frequent AI/ML components used for breast cancer (BC) detection and diagnostic.

This section of the survey outlines recent studies employing AI/ML techniques for the detection and categorization of breast cancer using various imaging methods, including mammography, ultrasound, magnetic resonance imaging (MRI), and computed tomography (CT). Using AI in the image analysis and management of breast cancer patients offers advantages related to early detection, diagnosis, and predicting the treatment response, which contributes to improved patient outcomes and the overall quality of breast cancer care. AI/ML learning methods rely on extracting hand-crafted features and employing one of the ML classifiers for detection or classification. The most commonly utilized AI/ML components for breast cancer (BC) diagnosis and detection, as shown in Figure 4, include SVM, decision tree (DT), random forest (RF), artificial neural network (ANN), autoencoder (AE), and CNNs.

### 6.1. Svm-Based Detection/Classification Methods

SVM possesses the capability to identify an optimal decision boundary that best represents the largest separation, or the widest margin, between different classes. Initially, it was developed to address problems involving linearly separable classes, but it was subsequently extended to handle non-linearly separable classes as well. SVM stands out as one of the most widely employed classifiers for the diagnosis and prediction of breast cancer (BC).

In the literature, various research groups have employed SVM for the detection and classification of breast cancer. For instance, Adel et al. [76] developed a technique to classify breast cancer into benign and malignant categories using B-mode ultrasound and elastography images. This approach involved extracting a total of 33 features from these images, including parameters such as width-to-height ratio, standard deviation, area, perimeter, mean, contrast-to-noise ratio, and signal-to-noise ratio. Principal component analysis (PCA) was applied to reduce the number of features from 33 to 18. Subsequently, SVM was employed for breast cancer classification, achieving an accuracy rate of 94.12%. Ara et al. [77] conducted a comparative study of various machine learning (ML) techniques for breast cancer classification into benign and malignant categories using the Wisconsin Breast Cancer Diagnostic (WBCD) dataset, obtained from the University of California Irvine (UCI) ML repository [78]. This dataset represents human breast tissue characteristics related to the size, shape, and texture of cell nuclei for each patient. Correlation analyses were performed on features such as radius, texture, area, and symmetry that characterize each category (benign vs. malignant), resulting in the elimination of less correlated WBCD features. For classification, different classifiers, including logistic regression (LR), SVM, RF, naïve Bayes (NB), DT, and k-Nearest Neighbors (KNN), were investigated. Ultimately, RF and SVM outperformed other classifiers, achieving accuracies of 96.5%.

In a study by Badr et al. [79], an optimized model served two purposes: (i) classifying breast cancer (BC) into benign and malignant using the WDBC dataset, and (ii) detecting BC records among healthy records using the Electronic Health Record (EHR) [80] dataset. Each EHR record comprised nine features: age, body mass index, glucose, insulin, homeostatic model assessment (HOMA), leptin, adiponectin, resistance, and monocyte chemoattractant protein-1 (MCP-1). Grey wolf optimization (GWO) was employed to determine optimal parameters for SVM classification. For data normalization, arithmetic, equilibration, and geometric mean scaling techniques were explored. Their method (GWO + SVM) with the equilibration scaling technique achieved the highest accuracy rate of 99.3% on the WDBC dataset (compared to a classical SVM accuracy of 82.05%) and 93.3% on the EHR dataset [80]. Khan et al. [81] applied a system for BC classification into malignant and benign using cytology images. Image pre-processing involved linear contrast enhancement and a linear filter for noise removal. After segmenting the cell objects using geometric active contours (GACs) to isolate cellular from non-cellular objects, the gray level co-occurrence matrix (GLCM) was computed from the segmented cell objects. Features such as contrast, energy, homogeneity, and entropy were computed from the GLCM matrix. Finally, an SVM classifier was employed to discriminate between malignant and benign cells, achieving an accuracy of 96.3%. Ed-daoudy and Maalmi [82] implemented a system for breast cancer (BC) classification into malignant and benign cases using the WBCD dataset. They reduced the initial nine features to either eight or four through the application of association rules (AR). When utilizing the eight selected features, an SVM classifier achieved the highest accuracy, reaching 98%. El-Azizy et al. [83] developed a computer-aided diagnosis (CAD) system designed to distinguish between malignant and benign nodules based on conventional B-mode ultrasound images. The CAD system comprises four phases: noise removal, lesion segmentation, feature extraction, and SVM classification. Firstly, they employed an anisotropic filter for noise reduction. Secondly, the active contour segmentation technique was utilized to delineate the tumors, either through a semi-automated or fully-automated approach. The initial active contour mask was determined either manually, by selecting a few points (semi-automated), or automatically using thresholding (fully automated). Subsequent to segmentation, three morphological features, namely perimeter, regularity variance, and circularity range ratio, were extracted from each lesion. Finally, SVM was employed for classification, resulting in accuracies of 95.98% (semi-automated) and 95.67% (fully automated, with a slight decrease in performance). Wei et al. [84] utilized breast ultrasound images to propose an automatic classification system for BC, distinguishing between malignant and benign nodules based on texture and morphological features. These features encompassed direct least-squares fitting of ellipses, compactness, and radial range spectrum, all extracted from manually defined regions of interest (ROI). SVM classifiers were employed for classification, yielding accuracies of 75.94% using only morphological features, 85.62% using solely texture features, and 87.32% when combining both texture and morphological features. Another study proposed by Rana et al. [85] introduced a system that used a microwave device to detect breast lesions. Initially, clinical data are extracted from patients using a microwave apparatus. The patient’s label, indicating whether their tissues are healthy or non-healthy, is determined through conventional breast exams such as echography, mammography, and magnetic resonance imaging. Subsequently, the clinical information (i.e., frequency domain signals) obtained through the microwave apparatus is input into three different classifiers in order to distinguish between normal and lesion tissues. The latter are KNN, Multilayer Perceptron (MLP), and SVM. Their results showed that the SVM demonstrated superior performance in breast cancer classification, achieving an accuracy rate of 98%, surpassing the performance of other methods. A similar study by Sami et al. [86] introduced a system for predicting breast lesions using microwave signals in the frequency domain, specifically S-parameters (S21). First, these frequency domain samples are subsequently transformed into time-domain signals through an inverse Fourier transform. Then, different machine learning algorithms were utilized to differentiate between healthy and non-healthy tissues by analyzing patterns found in the backscattered signals. Their results showed that the SVM with a third-degree polynomial kernel achieved an accuracy of 99.7%, surpassing the performance of traditional machine learning binary classification algorithms. These studies demonstrated the potential and effectiveness of integrating microwave signals with machine learning techniques for the early and accurate detection of breast lesions.

Table 7 provides a summary of various SVM-based breast cancer detection and diagnostic methods found in the literature. As illustrated in the table, diverse methodologies have been employed across various modalities and databases, encompassing ultrasound, elastography, cell tissue characteristics, patient records, cytology images, and more. These methods employ SVM classifiers that leverage distinct sets of extracted features, including statistical, appearance, morphological, and texture-based attributes. The outcomes obtained through these methodologies underscore the potential of incorporating AI/ML components to aid radiologists in breast cancer detection and diagnosis.

### 6.2. DT/Rf-Based Detection/Classification Methods

A predictive model known as decision trees (DT) in machine learning illustrates a mapping between object properties and object values. DT functions as a tree-like classifier, wherein each input data point can be categorized into specific classes based on each non-leaf node (representing a specific attribute) in a flowchart-like manner. Once the information gain has been estimated, a decision is made by determining the best path from the root node to a particular class (leaf). Some of the most widely recognized DT techniques, which employ entropy-based measurements for tree growth, include Iterative Dichotomiser 3 (ID3) [87], C4.5 [88], J48 [89], and classification and regression tree (CART) [90]. An ensemble model composed of multiple decision trees is commonly referred to as a Random Forest (RF) classifier. DT and RF stand out as popular classifiers for the diagnosis and prediction of Breast Cancer (BC).

In an experiment conducted by Singh et al. [91], various machine learning classifiers were explored for the classification of breast cancer into benign and malignant nodules. These classifiers included NB, binary logistic regression (BLR), and two DT classifiers, namely J48 [89] and the simple CART [90] classifiers. Their method underwent validation using the Wisconsin breast cancer original (WBCO) dataset, which was sourced from the UCI repository [78]. This dataset comprises a collection of characteristics pertaining to human breast tissues, including size, shape, and texture of cell nuclei. Manual preprocessing was executed to eliminate missing data values via a median filter. Among all the classifiers investigated, the simple CART classifier exhibited the highest accuracy, achieving 98.13%. Allada et al. [92] similarly delved into the examination of different machine learning classifiers such as KNN, SVM, DT, NB, LR, and RF for breast cancer classification using the WBCD dataset [78]. Preceding classifier training, preprocessing steps encompassed label encoding to convert categorical features into numerical ones, and feature value normalization within the range of 0 to 1. Among all the classifiers explored, both SVM and RF achieved the highest accuracy, registering at 96.5%. In the Sengar et al. [93] experiments, LR and DT were scrutinized for breast cancer classification using the WBCD dataset [78]. Data preprocessing involved label encoding and feature scaling applied to the WBCD features, with the DT classifier attaining a higher accuracy of 95.1%. The literature highlights the utilization of DT/RF-based techniques for breast cancer detection and diagnosis, as outlined in Table 8. This table underscores the competitive performance achieved by DT/RF classifiers in breast cancer classification.

### 6.3. ANN/AE-Based Detection/Classification Methods

The artificial neural network (ANN) is a machine learning model that draws inspiration from the capabilities and structure of biological neural networks. Within the realm of computer science, it exhibits functions akin to those of the human brain, encompassing tasks such as information reception, processing, and delivery. Owing to their competitive performance, ANNs have found application in breast cancer (BC) detection and classification. For instance, Abbass et al. [94] employed an evolutionary artificial neural network (EANN) based on the Pareto-differential evolution method (PDE) to predict BC. They conducted evaluations using the WBCD dataset [78], comparing their results with an evolutionary programming (EP) approach. Impressively, their EANN technique surpassed the EP approach with an accuracy rate of 98.12%. Similarly, Karabatak et al. [95] utilized the WBCD database [78], applying dimensionality reduction techniques to reduce the feature space from 9 to 4. Subsequently, they employed ANN for classification, achieving an accuracy of 95.6%. Furthermore, Jafari-Marandi et al. [96] developed a comprehensive framework for BC diagnosis, deploying it on both the WBCD and WDBC datasets, both sourced from UCI’s repository [78]. Their framework incorporated a self-organizing map (SOM) to project similarity and dissimilarity patterns among patients (benign and malignant) into a map that guided the training phase (error-driven learning) of a multi-layer perceptron (MLP). This approach resulted in accuracy rates of 96.2% on WDBC and 98.2% on WBCD. In a different approach, Rouhi et al. [97] tackled breast tumor classification into benign and malignant categories using two mammographic datasets: the mammographic image analysis society (MIAS) [98] dataset and the digital database for screening mammography (DDSM) [99]. Their methodology involved tumor segmentation using a cellular neural network segmentation, feature extraction from the segmented tumor regions, and feature selection via a genetic algorithm (GA). Subsequently, mammograms were classified using an ANN, achieving an accuracy of 90.16% on the MIAS dataset and 96.5% on DDSM.

When dealing with original datasets that lack labels and/or entail the expensive and challenging task of annotation, unsupervised learning emerges as an advantageous option. The primary objective of unsupervised learning is to fathom the underlying data structure, facilitating the extraction of valuable features. Among the prominent techniques in this realm is the AE, which autonomously encodes the initial input data into a lower-dimensional space representation, effectively compressing the data by leveraging an ANN as an approximating function. The compressed data subsequently finds utility in data reconstruction, with the aim of faithfully reproducing the original dataset. For simplicity, this task comprises two pivotal components: encoders and decoders. The encoder specializes in learning how to condense the original input into compressed data, while the decoder excels in the art of restoring the original data from the compressed counterpart. Autoencoders bear resemblance to PCA, yet they boast greater adaptability. Unlike PCA, which confines itself to linear transformations, autoencoders offer versatility by encoding data in both linear and non-linear manners. There exist four distinct types of autoencoders: (i) denoising autoencoders (DAE), which master the restoration of the unaltered input from partially corrupted input; (ii) sparse autoencoders (SAE), characterized by an architecture featuring more hidden encoding layers than input layers, sometimes employing the outputs of the final autoencoder as inputs for subsequent layers or within the broader network architecture, thus enabling the extraction of higher-level, abstract data representations through the gradual reduction and subsequent expansion of dimensionality; (iii) variational autoencoders (VAE), a unique autoencoder variant that integrates an additional loss component during training to approximate the posterior distribution in latent representation learning; and (iv) contractive autoencoders (CAE), which differ from standard autoencoders due to the incorporation of an explicit “regularizer” term in the training objective, promoting the model to acquire robustness against input data variations. In a study conducted by Kadam et al. (2019) [100], the authors employed an SAE-based approach, combined with softmax regression, for breast cancer (BC) classification into non-cancerous and cancerous cases, achieving an impressive accuracy of 98.59%. Table 9 highlights the application of ANN/AE-based BC detection and diagnostic methods found in the literature, showcasing their competitive performance in BC classification.

### 6.4. CNN-Based Detection/Classification Methods

CNN, an emerging type of ANN based on deep learning (DL), has garnered widespread recognition across various domains, including computer vision and medical fields. The primary feature of CNN lies in reducing the number of ANN parameters through parameter sharing and local processing, consequently diminishing computation complexity. The CNN architecture comprises three pivotal elements: convolutional layers, pooling layers, and fully connected layers. Training a CNN typically demands a substantial volume of training images, presenting a formidable obstacle in medical imaging due to the exorbitant cost of acquiring labeled datasets. To surmount this challenge, transfer learning has been introduced, leveraging pre-trained CNNs that were previously trained for other applications, thereby enabling the use of significantly smaller training databases. Transfer learning entails applying acquired knowledge from one task’s completion to another within the same domain or a related task. The benefit of deep learning, especially CNNs and transfer learning, lies in its ability to learn and extract features from large datasets, in contrast to machine learning, which relies on hand-crafted features. However, the disadvantages of using deep learning include model complexity, the need to tune a large number of parameters, the requirement for substantial computational resources for training on large datasets, and longer training times compared to machine learning.

In the literature, both basic CNNs and pre-trained CNNs are commonly employed for breast cancer (BC) detection and classification. For instance, Arevalo et al. [101] developed a CAD system for classifying mammography mass lesions as either malignant or benign. They applied their methodology to a dataset based on film mammography, sourced from the Breast Cancer Digital Repository (BCDR) [102]. Their CAD system consisted of three primary stages: preprocessing (comprising cropping, augmentation, and normalization), feature extraction (employing CNNs), and an SVM classifier, achieving an AUC of 83%. Zhang et al. [103] utilized a deep learning model for breast tumor classification into benign and malignant categories, based on features extracted from shear-wave elastography (SWE). Point-wise gated Boltzmann Machines (PGBM) and restricted Boltzmann Machines (RBM) were employed as a two-layer DL architecture for feature extraction. RBM was employed in an unsupervised pre-training phase to learn the input distribution’s probability, while PGBM combined feature selection and learning. The DL features extracted were subsequently input into an SVM classifier, achieving an accuracy of 93.4% for BC classification. Wang et al. [104] applied a DL model for benign/malignant BC classification using the Breast Cancer Histopathological Database (BreaKHis) [105]. They explored four magnification factors for histopathological images (40×, 100×, 200×, and 400×), utilizing a CNN to emphasize semantics and a capsule network to extract spatial information and other pertinent features. These convolutional and capsule features were subsequently merged via feature fusion and input into a modified capsule network for classification. The highest accuracy of 94.52% was attained using a 100× magnification factor. Ting et al. [106] adopted a DL approach for BC classification, categorizing patients into three classes: benign, malignant, and healthy, using mammographic images. They conducted feature-wise data augmentation and preprocessing, followed by employing a CNN for classification.

In a study conducted by Araújo et al. [107], a system was developed for classifying breast cancer histology using the Bioimaging 2015 breast histology classification challenge dataset [108]. Feature extraction was accomplished using a CNN, and for classification, an SVM was employed. To arrive at the final image classification, a patch-wise classifier was initially utilized to process several image patches. Subsequently, the classification results of all image patches were fused using three different methods: majority voting, maximum probability, and the sum of probabilities. Their system was capable of performing both multi-classification (categorizing normal tissue, benign lesions, in situ carcinoma, and invasive carcinoma) and binary classification (distinguishing carcinoma from non-carcinoma). The best results were obtained using majority voting, with a multi-classification accuracy of 77.8% and a binary classification accuracy of 83.3%. Kooi et al. [109] applied a deep learning approach to detect mammographic lesions, utilizing a local dataset collected from a screening program in the Netherlands. Handcrafted features, such as lesion location, contrast, context, texture, geometry, and patient age, were integrated with the CNN features to enhance the system’s performance, increasing it from 92.9% (when using only CNN features) to 94.1%.

Using the mini-MIAS [98] mammogram database, Tan et al. [110] employed abnormal tissue cropping and augmentation as preprocessing steps for a CNN model to classify mammogram images into three categories: normal, noncancerous abnormality, and cancerous abnormality. They achieved an accuracy of 82.71%, sensitivity of 82.68%, and specificity of 82.73%. In [111], Agnes et al. utilized a multiscale all CNN (MA-CNN) model to categorize mini-MIAS mammographic images into normal, malignant, and benign classes. Image preprocessing involved the application of a median filter for noise removal and global thresholding for artifact removal. Instead of employing a pooling operation, which can result in information loss, they opted for a larger stride convolution operation to reduce dimensions. To extract multiscale features, multiple dilated convolution operations were implemented, taking into account different receptive field sizes. Ultimately, they concatenated all feature maps generated by various receptive field sizes, followed by a convolution-stride before reaching the output layer. According to their experimental results, the MA-CNN model outperformed other tested CNN models in classifying mammogram images into normal, malignant, and benign categories.

Muduli et al. [112] employed a deep CNN methodology to automate the diagnosis of breast cancer across various mammography datasets (namely MAIS [98], DDSM [99], and INbreast [113]) as well as different ultrasound datasets (BUS-1 and BUS-2 [114]). Their methodology encompassed three key phases: preprocessing, deep CNN training, and classification. In the preprocessing phase, a manual cropping process was utilized to extract the ROI, followed by data augmentation through rotation, flipping, and scaling. To assess model stability and generality, they conducted five-fold cross-validation, repeated 10 times on diverse datasets, resulting in competitive performance in both mammogram and ultrasound images. In contrast, Haq et al. [115] harnessed a deep ensemble model for the classification of mammographic images into normal and abnormal categories. They applied unsharp masking to accentuate image edges and isolated the ROI using a Canny edge detector. Their CNN architecture comprised four major blocks, with the first three focused on feature extraction, while the final block consisted of a flattened layer, followed by three distinct parallel classifiers: sigmoid, SVM, and RF. To derive the ultimate prediction, a majority voting scheme was applied to the three classification responses. Their ensemble approach incorporated depth-wise convolution, spatial dropout, and data augmentation techniques.

In addition to the CNN models that were trained from scratch, transfer learning was frequently applied in breast cancer (BC) classification. For instance, Huynh et al. [116] employed transfer learning to classify mammographic images as benign or malignant. They combined two methods for classification: the first utilized a pre-trained CNN (AlexNet [117]) for feature extraction from the ROI and an SVM for classification. The second method extracted analytical features from the segmented lesion, including lesion size, shape, and margin characteristics such as speculation and sharpness. Subsequently, an SVM was applied to these features. The final classification was determined by soft voting, combining the outputs of the two SVM classifiers. Hu et al. [118] adopted a multi-parametric approach involving DCE-MRI and T2w-MRI. DCE-MRI provided complementary morphological and functional lesion information. They employed a pre-trained VGG19 network for feature extraction and training. Their approach involved combining data from DCE and T2w MRI sequences at three distinct stages: image fusion, feature fusion, and classifier fusion. For image fusion, an RGB composite image was generated from DCE and T2w images. For feature fusion, the features extracted from the VGG19 networks of each modality (DCE and T2w) were combined as input for the SVM classifier. Finally, classifier fusion was performed through soft voting, combining the output of the DCE and T2w SVM classifiers predicting malignancy probabilities. The feature fusion method statistically outperformed the other two fusion methods. For breast mass classification, Hassan et al. [119] employed two pre-trained CNN networks: AlexNet [117] and GoogleNet [120]. Mammogram images were pre-processed using the maximally stable extremal regions (MSER) [121] method. These CNN networks were trained and tested on mammogram images from CBIS-DDSM [122] and INbreast [113] databases, and they were also tested on the MIAS database [98] and real cases from the Egyptian National Cancer Institute. Their results demonstrated that the AlexNet model outperformed the GoogleNet model in BC classification.

Wang et al. [123] employed a modified Inception V3 network [124] to distinguish between malignant and benign lesions in ultrasound images captured from two perspectives: coronal and transverse. They conducted four experiments, with two relying on a single view and the remaining two utilizing multi-views. In all experiments, their adapted Inception V3 model was utilized for feature extraction. In the initial two experiments, features were extracted exclusively from either the coronal or transverse view of the cropped lesion. In the third experiment (referred to as CNN A), they concatenated the two views of the same cropped lesion (transverse and coronal) and fed them into a modified Inception V3 model for classification. In the last experiment (referred to as CNN B), two Inception-v3 models were employed, one for each specific view. Features generated from these two Inception models were subsequently concatenated into a final layer for classification. The experiments were evaluated using data collected at the Jeonbuk National University Hospital (JNUH), where CNN A achieved the highest performance. Meanwhile, to classify benign and malignant mammogram structures, including benign masses, malignant masses, benign calcifications, and malignant calcifications, Hekal et al. [125] applied optimal Otsu thresholding [126] to segment suspected nodule regions. These segmented regions were further processed using either AlexNet or ResNet-50, and an SVM was then employed for the classification task. In a subsequent work, Hekal et al. [127] utilized an ensemble comprising four CNN models (ResNet-50, ResNet-101, AlexNet, and DenseNet-201) to process suspected nodule regions segmented through automated thresholding, aiming to classify benign and malignant mammogram structures.

A recent study by Moreau et al. [128] proposed an automatic segmentation system to detect breast cancer metastatic lesions on longitudinal whole-body PET/CT. First, the authors used a U-Net network to segment baseline images and follow-up images. Then, four different biomarkers were extracted from these segmentations to evaluate how patients respond to their treatment. The latter are SUL ${}_{peak}$, total lesion glycolysis (TLG), PET bone index (PBI) and PET liver index (PLI). Their results showed that SULPeak is the most effective biomarker in evaluating patients’ response with a sensitivity and specificity equal to 87%.

The discussed CNN-based detection and diagnostic techniques are listed in Table 10. As seen in the table, various DL techniques such as augmentation, spatial drop-out, transfer learning, fusion, ensemble learning, etc., were employed across various modalities and databases including US, mammogram, elastography, histopathology, DEC-MRI, T2w-MRI, and multi-parametric data. The outcomes of these techniques underscore the potential of employing DL and CNN models to aid radiologists in breast cancer detection and diagnosis. Table 11 summarizes the most frequently utilized modalities, features, and AI/ML components in the breast cancer detection and diagnosis literature. This literature demonstrates how these AI/ML components play a crucial role in providing objective quantitative metrics for breast cancer identification and diagnosis, potentially enhancing the quality of healthcare systems with regard to breast cancer.

## 7. Molecular Breast Cancer Subtypes and Imaging Techniques

Breast cancer is a diverse disease encompassing various molecular subtypes that can profoundly impact prognosis. Recent advancements in non-invasive imaging techniques, such as Molecular Breast Imaging (MBI), have emerged to predict these subtypes.

### 7.1. Molecular Breast Cancer Subtypes

Molecular classifications have opened the door to understanding that breast cancer (BC) is not a uniform disease [130]. The breast molecular subtype proves to be a reliable prognostic factor for survival as it correlates with tumor aggressiveness [131]. According to immunohistochemical markers such as the estrogen receptor (ER), progesterone receptor (PR), human epidermal growth factor receptor 2 (HER2) status, and Ki-67 expression, four distinct molecular subtypes of BC have been established, each characterized by unique gene expression profiles. This classification also significantly impacts clinical outcomes and the response to treatment [132]. These four subtypes are:

- Luminal A: positive ER and PR, negative Her2, and low proliferation index.
- Luminal B: positive ER, and either positive Her2 or high proliferation index.
- Her 2 enriched: negative ER and PR, and positive Her2.
- Triple-negative: negative ER, PR, and Her2.

Most invasive breast cancers are classified into luminal A and B groups, which are associated with better survival rates. The HER2-enriched subtype, comprising 10% to 20% of breast cancers, responds well to HER2-directed therapy [133]. However, HER2-enriched cancers exhibit superior responses to chemotherapy but exhibit poorer overall and disease-free survival outcomes [134]. Triple-negative breast cancer represents approximately 15% to 20% of all invasive breast cancer cases [135]. Patients diagnosed with the triple-negative subtype typically experience worse prognoses and a higher likelihood of recurrence [136].

### 7.2. Molecular Breast Imaging (MBI)

Molecular imaging techniques, such as PET-CT and PEM, provide quantitative biomarkers that convey valuable information about tumor receptor status, the extent of tumor diversity, and the response to treatment [137]. MBI successfully addresses the limitations of tissue-based biomarkers by enabling noninvasive evaluation of the entire body, either singly or multiple times. Furthermore, MBI serves as a problem-solving tool for assessing complex mammography or unexplained physical findings [138]. In this section, we will provide an overview of various MBIs and their applications in breast cancer.

#### 7.2.1. PET-CT

**Figure 5:**
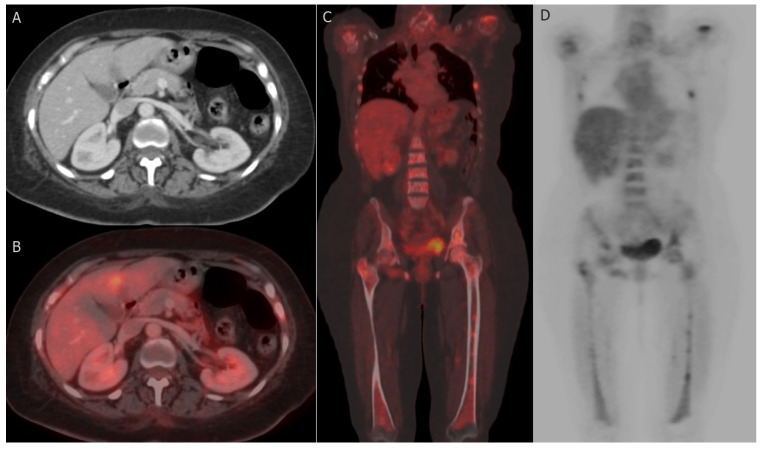
Hepatic and bony metastases: 61-year-old woman with grade II invasive ductal carcinoma who underwent a right modified radical mastectomy. (**A**,**B**) axial post-contrast CT image and fused FDG PET/CT image reveal FDG-avid hepatic metastatic deposit at segment IV b with a maximum standardized uptake value (SUVmax) of 8.5. (**C**,**D**) coronal-fused FDG PET/CT and maximum intensity projection (MIP) images reveal FDG-avid multiple bony metastatic deposits at ribs, proximal humeri, lumbar vertebrae, iliac bones, and both femori with SUVmax of 9.8.

PET-CT, utilizing the radiotracer 18F-Fluorodeoxyglucose (18F-FDG), has proven to be a dependable non-invasive imaging modality for distinguishing benign from malignant lesions, offering substantial benefits in assessing tumor response [139]. FDG PET-CT can effectively identify malignant breast masses, as tumor cells exhibit elevated glycolytic activity and increased FDG uptake, which may also be influenced by the tumor grade [140]. PET-CT provides comprehensive metabolic and morphologic information, along with quantitative data regarding tumor activity [141]. The most commonly used method for quantifying FDG avidity is the standardized uptake value (SUV), available in various forms depending on the ROI considered, such as maximum, mean, or peak SUV [142]. Both SUVmax (maximum standardized uptake value) and metabolic tumor volume are more reliable and reproducible quantitative parameters compared to measuring tumor size [143]. Although PET-CT exhibits lower sensitivity in diagnosing primary breast cancer when compared to specialized breast imaging methods like mammography, ultrasound (US), and breast MRI, it plays a pivotal role in systemic staging and the detection of tumor response and recurrence [144] (Figure 5). The medical applications of PET-CT are further illustrated as follows:

**Diagnosis:** The National Comprehensive Cancer Network (NCCN) guidelines do not endorse the routine use of PET-CT for the initial diagnosis of breast cancer [139]. However, PET-CT may offer advantages, particularly in the initial staging of patients at a substantial risk of developing metastasis [145]. Progress in dedicated breast imaging techniques, such as positron emission mammography (PEM), has enhanced the nuclear medicine assessment of primary breast lesions [146].

**Prognosis:** PET-CT offers distinct advantages over conventional imaging modalities when it comes to delivering prognostic stratification. Unlike conventional methods that solely assess the morphological features of the primary tumor [147], PET-CT has gained approval for its ability to aid in risk classification for advanced stage breast cancer (BC) [148,149]. Notably, FDG uptake demonstrates a significant correlation with tumor grade, aggressiveness, and overall prognosis [150]. Recent research findings have revealed a positive association between SUVmax values and various factors, including tumor size, clinical stage, specific molecular subtypes (such as the triple-negative subtype), and the Ki-67 index [151]. Specifically, the triple-negative subtype exhibits markedly higher FDG uptake compared to luminal subtypes, while the luminal B subtype shows significantly higher FDG uptake than the luminal A subtype [152]. High initial pretreatment SUVmax values are predictive of poorer outcomes in specific BC types, notably the luminal type and invasive ductal carcinoma (IDC) [153]. Furthermore, a higher SUVmax may indicate an elevated risk of recurrence, particularly among patients with hormone receptor-positive breast cancer [154]. However, it is worth noting that pretreatment SUVmax may have limited utility for tumors with lower FDG avidity, such as lobular carcinoma [155].

**Nodal metastases:** The most crucial prognostic indicator influencing the treatment strategy for breast cancer (BC) is the presence of lymph node (LN) metastases, as highlighted in a study by Mohammed et al. [156]. Historically, surgery served as the gold standard for obtaining LN staging information. However, the landscape has evolved with the increased utilization of neoadjuvant systemic therapy and a preference for less extensive surgical interventions whenever feasible. Consequently, the role of radiologic staging has gained prominence in recent years, as emphasized by Chung et al. [157]. It is important to note that locoregional nodal metastases encompass both axillary lymph nodes (ALN) and extra-axillary LNs, as elucidated by Ulaner et al. [141].

i. *Axillary nodal metastasis:* When staging axillary lymph nodes (ALNs), sentinel node biopsy (SNB) remains the gold standard [158]. It is defined as the initial site to receive breast lymphatic drainage and represents the primary location for ALN infiltration [159,160]. This sentinel node can be identified using various methods, including blue dye, radioisotopes, ICG (indocyanine green), or their combination, and subsequently retrieved intraoperatively for histopathological examination to determine the necessity for ALN dissection [160]. In contrast to SNB, FDG PET-CT exhibits reduced sensitivity in detecting axillary lymph node (ALN) metastases [161,162]. Nevertheless, FDG PET-CT has shown comparable performance to other non-invasive imaging modalities such as ultrasound (US) and MRI for ALN detection [149]. In a previous study, PET-CT demonstrated notably higher accuracy than ultrasound (US) [163]. It is worth noting that PET-CT has better specificity than sensitivity for detecting ALN metastasis, particularly in early-stage cases [164]. SUVmax may serve as a potential prognostic factor for axillary lymph node (ALN) metastases, especially in specific breast cancer subtypes like HER2-positive and ER-positive/HER2-negative tumors [130].
ii. *Extra-axillary nodal metastasis:* Regional extra-axillary lymph nodes, which encompass the internal mammary, infraclavicular, and supraclavicular lymph nodes, are less frequently identified through sentinel node assessment [141]. FDG PET-CT offers superior accuracy in staging by detecting extraaxillary nodal metastases, particularly excelling over ultrasound in the detection of internal mammary nodal involvement [165,166]. The discovery of unexpected metastatic lymph nodes beyond the axillary region during the initial staging using FDG PET-CT has a profound impact on patient prognosis and can potentially influence decisions regarding the extent of surgical or radiotherapeutic interventions [167].

**Distant metastases:** The conventional imaging modalities for detecting distant metastasis in BC include anatomic imaging with contrast-enhanced CT, bone scintigraphy, and MRI. More recently, functional imaging with FDG-PET/CT has been performed [147]. FDG-PET/CT is recommended for initial staging in patients with clinical stage ≥ IIB BC [168]. The most common sites of distant metastasis in BC are bones, lungs, liver, and brain [169].The functional advantage of PET-CT permits detection of early metastasis in the bone, the most common site of metastasis, which may stay undetected with bone scintigraphy [170]. PET/CT is furthermore efficient in detecting extra-skeletal metastases, comprising, pleural, hepatic, splenic, and pelvic metastases [145,149,171]. A recent comprehensive literature review confirmed that PET/CT is very efficient in identifying occult distant metastases (except for brain), with superior performances compared to those of conventional imaging [171]. In a recent prospective study of 103 BC patients, 24 (23%) were diagnosed with distant metastases by FDG-PET/CT. Owing to these findings, breast surgery was cancelled in 18 while the other 16 patients were upstaged, leading to more extensive radiotherapy. So, they concluded that FDG-PET/CT should be considered for primary staging in high-risk BC to improve management planning [172].

The new development of PET tracers that act as fibroblast-activation-protein inhibitors (FAPIs) exhibited promising results, FAP is overexpressed by tumour-associated fibroblasts of various tumors [173]. A recent prospective comparative study of 34 newly diagnosed BC patients concluded that the 68Ga-FAPI SUVmax was positively correlated with the pathological grade and the final stage of the patients. Also, 68Ga-FAPI PET/CT revealed higher accuracy than 18F-FDG in the evaluation of N stage, which may improve the treatment strategy [174]. A few recent case reports and small pilot studies highlighted the role of 68Ga-FAPI PET/CT in detection metastases in BC, which need to be confirmed by further larger studies [175,176].

#### 7.2.2. Positron Emission Mammography (PEM)

Positron emission mammography (PEM) is a recent breast-specific technology that offers high-resolution detection of 18-FFDG uptake, producing images equivalent to those utilized in mammography. This capability allows for convenient image comparison [177]. PEM holds a significant advantage over PET-CT due to its superior spatial resolution, particularly for detecting small and low-grade lesions, boasting an overall sensitivity of 91% and specificity of 93% [178]. PEM excels in detecting tumors as small as 2 mm, whereas whole-body PET-CT struggles with breast cancers smaller than 10 mm [146]. Typically, PEM is primarily employed for staging and preoperative planning, especially when MRI is contraindicated [179]. Furthermore, PEM plays a vital role in enhancing the management of women with mammographically suspicious microcalcifications, as it can detect invasive carcinomas and high-grade ductal carcinoma in situ (DCIS), thereby preventing unnecessary biopsies in benign cases [180]. Additionally, PEM finds use in distinguishing recurrent tumors from scars and evaluating responses to neoadjuvant chemotherapy [181].

## 8. Breast Cancer Imaging Biomarkers

In the current advanced era of precision medicine, there is an augmented need to integrate breast imaging with correlated biomedical disciplines to create comprehensive databases encompassing clinical, laboratory, and imaging biomarkers, ultimately improving breast cancer management [182]. For many years, breast cancer treatment has relied on tissue-based biomarkers, which involve assessing the expression levels of ER, PR, HER2, and Ki-67 [183]. However, tissue-based biomarkers have limitations in detecting the diverse characteristics of breast cancer within both the primary tumor and its metastatic sites. Furthermore, assessing the evolving features of metastatic breast cancer over time using these biomarkers is complex, necessitating successive biopsies [184]. Recognizing the critical diagnostic and predictive roles of imaging, biomarkers are now considered measurable indicators of biological processes obtainable from either tissue or imaging [137]. Imaging-based biomarkers encompass diagnostic, prognostic, predictive, and pharmacodynamic categories, making it crucial to distinguish among these types when discussing their clinical significance [185].

Diagnostic biomarkers are employed for disease verification and the detection of its specific subtype, while pharmacodynamics biomarkers evaluate the impact of systemic therapy or intervention. This outcome may not necessarily correlate with a positive result [186]. Conversely, predictive biomarkers aid in selecting optimal therapies for patient care by identifying individuals more likely to respond favorably or unfavorably to specific treatments compared to those without the biomarker [182,187]. Prognostic markers indicate disease progression or recurrence and can assess the inherent prognosis of the disease, but they do not provide guidance for treatment decisions [188]. Table 12 enumerates examples of imaging-based breast biomarkers.

Certain biomarkers can fall into multiple categories depending on the clinical question. For example, the maximum standardized uptake value (SUVmax) derived from 18F-FDG PET-CT scans can serve as predictive or pharmacodynamic biomarkers [189,190]. Additionally, ER, PR, and HER2 may provide diagnostic, predictive, or prognostic biomarkers [182].

## 9. Management of Breast Cancer

Breast cancer management is a multifaceted endeavor, relying on five primary treatment modalities: surgery, radiotherapy, chemotherapy, hormonal therapy, and targeted therapy [191]. The customization of each patient’s treatment plan hinges primarily on the disease stage and the molecular profile [192]. In this era of personalized medicine, intricate patient-specific details play a pivotal role. These encompass decisions regarding which treatment line is most suitable, the timing of interventions, and the sequence of these therapeutic approaches. The administration of these treatment modalities within specialized, high-volume breast cancer centers, guided by multidisciplinary team assessments, is no longer an extravagance but a determinant of patients’ oncological outcomes and their overall quality of life [192]. Surgery encompasses both breast and axillary procedures. Within breast surgery, there are two primary categories: breast-conserving surgery and mastectomy, the latter of which may or may not involve reconstruction. Breast-conserving surgery represents the gold standard for early-stage breast cancer [193]. Mastectomy, on the other hand, remains a vital alternative for those ineligible for breast conservation, such as the patients with locally advanced tumors at the time of surgery, patients unsuitable for breast irradiation, individuals with multicentric tumors that cannot be sufficiently removed through oncological resection, and those who opt against breast conservation [192,193,194,195,196]. For patients undergoing mastectomy, breast reconstruction surgeries, whether involving synthetic or autologous implants, offer crucial options [197,198,199]. Axillary surgery encompasses procedures such as axillary lymph node dissection (ALND) and sentinel lymph node biopsy (SNB). SNB has become the standard approach for node-negative breast cancer and selected cases of node-positive breast cancer. However, axillary dissection remains necessary for patients with multiple metastatic axillary lymph nodes at the time of surgery, as well as for individuals who are not suitable candidates or have experienced failed sentinel lymph node localization [160,200,201,202,203].

The other local treatment modality is radiotherapy, which is indicated following breast-conserving surgery or in patients who have undergone mastectomy with a large tumor size or heavy axillary disease. Radiotherapy can be administered in two main forms: conventional external beam radiation or partial breast irradiation. The latter includes localized conformal external beam radiation therapy, brachytherapy, and intraoperative single-fraction (IORT) treatment [192,204,205,206]. Systemic treatment encompasses three additional approaches: chemotherapy, hormonal therapy, and targeted therapy, which can be administered either in the adjuvant or neoadjuvant context. Neoadjuvant therapy is recommended for patients with locally advanced cancer or early-stage breast cancer, with the aim of enabling breast preservation or reducing lymph node involvement to make sentinel lymph node biopsy (SNB) an option instead of axillary lymph node dissection (ALND). Notably, it has become the standard practice for aggressive breast cancer subtypes such as triple negative and HER2-enriched, even in the early stages of the disease [192,200,207,208,209]. Chemotherapy is indicated in the adjuvant setting for patients with malignant lymph nodes, invasive tumors larger than 0.5 cm (except for luminal A type), and in luminal A breast cancer if the Oncotype DX score is greater than 31. It should be administered within 6 weeks of the operation, typically involving a combination of Anthracycline-based and Taxane-based chemotherapy for 6–8 cycles [200,210,211]. Hormonal treatment is recommended for luminal breast cancer, using anti-estrogens, aromatase enzyme inhibitors, and ovarian suppression or ablation [200,212]. Targeted therapy (anti-HER2 therapy) is employed for patients with HER2 overexpression (HER2-enriched or luminal B-HER2+ type), improving response rates and patient survival when combined with taxane chemotherapy [133,134].

## 10. Assessment of Treatment Response

BC employed a range of treatment strategies tailored to variable prognostic factors, which encompassed factors such as tumor stage, nodal metastases, and molecular subtype. Precise evaluation post-treatment was imperative for both locally advanced and metastatic BC cases.

### 10.1. Assessment of Neoadjuvant Therapy Response

Neoadjuvant chemotherapy (NAC) is no longer limited to treating locally advanced BC; more recently, it has been employed to downstage the disease, facilitating conservative surgery or avoiding axillary nodal dissection [158]. The response to NAC is frequently evaluated using breast MRI and, to a lesser extent, ultrasound (US), mammography, and clinical examination, to distinguish between responders and non-responders [213,214]. MRI surpasses ultrasound (US) in accurately determining tumor size before surgery following NAC [215]. Monitoring axillary lymph nodes (ALN) and tumor size using both US and MRI is valuable for predicting axillary response to NAC, with the highest diagnostic performance achieved by US during NAC [216]. Additionally, a different study demonstrated that ultrasound (US) evaluation of ALN following NAC showed the strongest independent association with the presence of residual axillary metastasis during surgical procedures [217].

Higher baseline FDG activity and a greater decrease in SUVmax after the early cycles of NAC may indicate improved histopathological status following NAC [218]. Previous studies have consistently shown a strong association between early changes in SUVmax and NAC response, as assessed through pathological examination [219,220]. More recent studies have proposed principles for predicting NAC response in various tumor subtypes based on FDG activity measures [135,174]. A meta-analysis study has demonstrated that PET/CT has moderate accuracy in predicting pathological response during the early cycles of NAC in breast cancer patients, and it suggests further prospective studies to better understand PET/CT’s role in evaluating NAC response [221]. However, no imaging modality has shown the ability to differentiate partial response from complete response, as low-volume residual disease may persist despite no evidence on imaging [214,222]. Radiological complete response by MRI cannot accurately predict pathological complete response (pCR) after NAC, making pathologic assessment of the breast tumor and axillary lymph nodes necessary [223,224]. A recent study found that the effectiveness of MRI in predicting complete pathological response varied among molecular subtypes, with the HR/HER2+ subtype having the highest rate of false-negative results [225]. However, MRI-detected residual lesions can consistently indicate non-pCR in the luminal subtype [226]. To achieve accurate predictions of pCR, the study recommends combining PET/CT and MRI [227].

### 10.2. Assessment of Response in Metastatic Breast Cancer

The standard for assessing treatment response in metastatic breast cancer relies on tumor size measurements, typically via CT scans [228]. Molecular breast imaging (MBI) outperforms anatomic changes in detecting tumor response, as it can gauge metabolic alterations, especially in the presence of therapy or surgery-induced anatomical changes [147]. There are fewer published studies evaluating treatment response in metastatic breast cancer compared to those investigating responses to NAC. This disparity arises because while pathologic examination is typically available as a reference standard after NAC, it is rarely accessible following treatment for metastases [141]. Previous research has demonstrated that PET-CT serves as a clinically significant biomarker capable of distinguishing response from non-response in metastatic breast cancer [229]. PET-CT surpasses CT in its ability to detect treatment responses in bony metastases because changes in bone density visible on CT scans after treatment may indicate bone healing rather than the emergence of new metastases. Consequently, the utilization of PET-CT helps prevent inaccurate evaluations of treatment response solely relying on CT scans [230,231]. Few studies have compared the diagnostic performance of both CT and PET-CT in assessing treatment response in metastatic breast cancer, revealing that PET-CT was a superior predictor of both disease-specific and progression-free survival compared to CT [232]. Monitoring treatment response with PET-CT in metastatic breast cancer has the potential to enhance patient management, although further research is required.

### 10.3. The Role of AI in the Assessment of Treatment Response

The utilization of AI/ML approaches for predicting early responses to neoadjuvant chemotherapy (NAC) holds the promise of improving precision in anticipating the probability of achieving a pCR before the commencement of treatment. Machine learning methodologies are particularly adept at constructing models that integrate clinical and imaging data, given their ability to effectively handle and develop models from vast and intricate datasets. Researchers have initiated investigations into the use of AI/ML methods employing imaging data to assess the responses of breast cancer (BC) patients to NAC treatment. For example, Mani et al. [233] introduced an approach to assess BC response to NAC, classifying it into two categories: pCR and non-responders. A pCR is characterized by the complete elimination of all invasive cancer within the breast following the conclusion of NAC [234], while non-responders are defined as cases where no change occurs in the lesion after NAC. They utilized quantitative MRI techniques such as DCE-MRI and DWI-MRI, rather than conventional MRI, to generate thirteen imaging features in conjunction with standard clinical information. Several ML algorithms were employed, including three linear classifiers (Gaussian Naïve Bayes (GNB), LR, and Bayesian logistic regression (BLR)), and two decision tree-based classifiers (CART and RF). Additionally, Gram-Schmidt orthogonalization with a selection of ten features (GS-10) was used as a feature selection method. Using a cohort of 20 patients collected at Vanderbilt University Medical Center, Nashville, TN, USA, the study reported that the combination of imaging (DCE-MRI and DWI-MRI) and clinical factors significantly improved the performance of BLR, resulting in an accuracy of 0.9 and an AUC of 0.96, even without the use of feature selection (GS-10).

Tahmassebi et al. [235] employed an approach to assess the response of breast cancer (BC) to NAC by categorizing it into four residual cancer burden (RCB) classes: RCB 0, indicating the absence of any invasive cancer in the breast after NAC completion; RCB 1, representing a small amount of residual disease; RCB 2, indicating a moderate residual disease burden; and RCB 3, signifying no change in the lesion following NAC. Furthermore, they aimed to predict survival outcomes, specifically disease-specific survival (DSS) and recurrence-free survival (RFS), in BC patients. They utilized both qualitative and quantitative features extracted from multiparametric MRI, encompassing T2-weighted MRI, dynamic contrast-enhanced MRI (DCE-MRI), and diffusion-weighted MRI (DWI-MRI), to forecast the response of breast cancer to NAC. Eight different classifiers were employed, including linear discriminant analysis (LDA), SVM, LR, RF, stochastic gradient descent (SGD), DT, AdaBoost, and XGBoost. Ultimately, in a cohort of 38 patients, they demonstrated that the XGBoost classifier outperformed all other classifiers, achieving an AUC of 0.94 for predicting pCR and an AUC of 0.92 for predicting DSS. However, the LR classifier slightly outperformed XGBoost in RFS prediction, with an AUC of 0.86. Bhardwaj et al. [236] employed a framework to assess the response of breast cancer, utilizing a dataset consisting of 222 subjects from the breast imaging research project [237]. They utilized three ensemble models, namely LR, AdaBoost, and Adabag, for prediction and employed stacking instead of majority voting to produce the final prediction. For performance evaluation, they calculated the weighted simple additive weighting (WSAW) score using ten evaluation criteria: true positive rate (TPR), false positive rate (FPR), precision, recall, F-Measure, Matthews correlation coefficient (MCC), accuracy, mean absolute error, root mean square error, and AUC. Using this dataset, their framework outperformed other classification models, including Bayes Net, RF, Adaboost, Adabag, and NB, with an accuracy of 99.1%.

Aghaei et al. [238] introduced two methodologies for the early prediction of tumor response to NAC, classifying it into two categories: complete response and partial response with no response. They extracted 39 kinetic image features organized into five groups from both the tumor and background parenchymal enhancement regions, encompassing the tumor area, enhanced area, necrotic area, and background parenchymal area. In the first methodology, they evaluated the discriminative potential of each image feature using ROC curves and identified non-redundant features by analyzing correlation coefficients. Subsequently, they devised a novel categorization score through a straightforward fusion procedure, serving as a predictor for the tumor’s response to NAC. The second methodology selected 11 characteristics and employed an ANN as a classifier, using a wrapper subset evaluator (WSE). Evaluation was conducted using data from 68 breast cancer patients, employing a leave-one-case-out approach, where the ANN method outperformed the simple fusion method with an impressive AUC of 0.96. Sutton et al. [239] adopted an approach for predicting tumor response to NAC, categorizing it into two classes: pCR and no-response (no-pCR). They amalgamated radiomics features derived from T1-weighted fat-saturated pre-contrast and three post-contrast MRI sequences with molecular subtypes (Luminal A, Luminal B, HER2+, and Triple-negative). They subsequently performed feature selection using maximum relevance minimum redundancy (MRMR) and generalized linear regression with elastic net (GLMNet). Finally, they employed recursive feature elimination with RF (RFE-RF) as a classifier, evaluating it using data from 273 patients. The combination of radiomics features with molecular subtypes notably improved prediction performance, elevating the AUC from 0.72 to 0.80. Vicent et al. [240] harnessed six machine learning algorithms (KNN, DT, RF, AdaBoost, GBoost, GNB, LDA, LR, and MLP) to predict tumor response to NAC, classifying it into two categories: pCR and no-pCR. They assessed their algorithms using data from 58 patients collected at Castellón provincial hospital, Spain. Their study demonstrated that integrating radiomics features and imaging features extracted from DWI-MRI and DCE-MRI, such as gray-level size-zone matrix, GLCM, and gray-level run length matrix, along with clinical data such as molecular subtype and clinical tumor stage, significantly enhanced performance compared to using clinical or imaging features alone, achieving an accuracy of 91.5%. Collectively, the literature studies presented [233,235,236,238,239,240] underscore the potential for improving the pre-treatment prediction of tumor response to NAC through the application of AI/ML techniques.

## 11. Conclusions

Artificial intelligence (AI) has seamlessly integrated itself into the medical field, with a significant impact on diagnostic imaging, where continuous developments in AI technology have led to its widespread adoption across various medical applications. In the realm of breast cancer (BC), deep learning techniques have found extensive application, facilitating automated diagnosis, segmentation, data analysis, and outcome predictions. Recent studies have showcased encouraging outcomes by harnessing deep learning algorithms for BC diagnosis and precise feature segmentation, thereby underscoring AI’s potential to enhance the accuracy and efficiency of BC diagnosis and analysis. To encapsulate the key findings of this survey:

- Structured BI-RADS reports provide assessment categories that encompass breast density, a description of detected findings, and recommendations for managing the identified abnormalities [8].
- Digital Mammography (DM) is the ideal method for screening and early detection of BC, but it has low sensitivity in dense breasts [28].
- Updated mammographic modalities such as digital breast tomosynthesis (DBT) and contrast-enhanced mammography (CEM) are proposed to overcome this shortage [32,33].
- Breast US lexicon has been updated to reflect advanced techniques such as elastography. Also, the “special cases” category has been extended in the BI-RADS 5th edition [7].
- Currently, MRI is the key technique for imaging breast cancer with the highest sensitivity (88–100%) among breast imaging modalities [49].
- Molecular classifications opened the door to understanding that BC is not a uniform disease. The molecular subtype affects the clinical outcomes and the response to treatment [130].
- MBI offers quantitative biomarkers, which indicate tumor receptor status, tumor aggressiveness, and treatment response [137].
- PET-CT has a critical role in systemic staging and the detection of tumor response and recurrence of BC, but PET-CT has low sensitivity to diagnose primary BC compared to other dedicated breast imaging [144].
- PEM has a great advantage over PET-CT owing to its higher spatial resolution, particularly for small and low-grade lesions, with overall 91% sensitivity and 93% specificity [178].
- Radiologists must be familiar with variable BC imaging biomarkers [185].
- Breast cancer management is multimodal depending mainly on the disease stage and the molecular profile [191].
- Response to NAC is frequently assessed by breast MRI and, to a minor extent, US to discriminate NAC response from nonresponse. MRI is superior to US in preoperative tumor size assessment after NAC [213].
- The evaluation of treatment response in metastatic breast cancer commonly relies on measuring tumor size, typically using CT scans [228].
- Utilizing ML classifiers using different extracted features (e.g., statistical [241,242,243], appearance [241,242,243,244,245,246,247,248,249], morphological [241,242,243,246,247,248,249,250], texture [241,242,243,244,245,246,250,251,252,253,254,255], etc.), various investigated methods were applied to various modalities/databases (e.g., ultrasound, elastography, cell tissue characteristics, patient records, cytology images, etc.). The outcomes of these ML-based techniques highlight the potential of utilizing ML classifiers for BC detection and diagnosis [76,81,92].
- On various modalities/databases (e.g., US, mammography, elastography, histopathology, DEC-MRI, T2w-MRI, multi-parametric data, etc.), various DL technologies (e.g., augmentation, spatial drop-out, transfer learning, fusion, ensemble learning, etc.) were utilized. The results of these DL methods demonstrate the possibility of using CNN and DL models to assist radiologists in BC identification and/or diagnosis [100,118].
- The fusion of the extracted AI features from multiparametric modalities can improve the performance of BC classification [118].
- ML/AI components are able to provide quantifiable, objective measures for BC detection and diagnosis and can help with pre-treatment tumor response prediction to NAC. Therefore, their findings have the potential to enhance the effectiveness of the healthcare systems for BC [233,235,236,238,239,240].

The future holds advances in technology, which can be outlined as follows:

- There is a need for an updated BI-RADS lexicon for the proper application of evolving imaging modalities, such as contrast-enhanced mammography and molecular breast imaging (MBI).
- Further investigations into the role of DTI in BC diagnosis are required.
- Monitoring treatment response with PET-CT in metastatic BC may improve metastatic patient management, however further investigation is needed.
- The recent advances in AI/ML (i.e., DL techniques, transfer learning, ensemble learning, etc.) have the potential to effectively improve healthcare outcomes for BC detection, diagnosis, classification, and treatment prediction [118,119,123].
- Further investigation for utilizing AI/ML CAD systems based on alternative nonionized modalities (other than the ionized mammograms) should be explored to reach acceptable clinical performance [86].
- Constructing large standard online databases for the purpose of evaluating developed AI-based systems for BC detection, diagnosis, classification, and/or treatment prediction can help to evolve the evolution of AI in this field.

Overall, this survey overviews updated information about the BC molecular subtypes, advanced imaging techniques, tumor response assessment, and variable treatment strategies that improves the radiologists’ role in the tailored care of BC patients. BI-RADS is expected to continue to develop for application in a variety of evolving imaging modalities. Details regarding BC molecular subtypes, biomarkers, molecular imaging, and the promising role of AI are shown in this review to provide a source of updated knowledge and further research.

## Figures and Tables

**Table 1:** Assessment categories and guidance in the 5th edition of BI-RADS. ACR and SBI stand for American College of Radiology and Society of Breast Imaging, respectively [8].

Category	Description	Recommendation
0	Incomplete assessment: a. further examination is required b. comparison films requested	a. Supplementary assessment with mammography, US, or less frequently MRI; after accomplishment of workup, further final category is offered. b. Comparison can only be applied when it is necessary to obtain a final assessment.
1	Negative: negative assessment	Screening within 1 year (per SBI and ACR recommendations); no expected malignancy.
2	Benign findings: to express a benign lesion that has no malignant possibility	Screening within 1 year (per SBI and ACR recommendations); similar to category 1. Category 1 is favored over category 2 whenever suitable to avoid patient and clinician anxiety and requesting unnecessary imaging examinations after the description of benign findings.
3	Probably benign finding: debatable category applied when a finding is nearly definitely benign but preferred to have a short interval follow-up; unlikely to demand biopsy. It holds a risk of malignancy up to 2%.	Short interval follow-up examinations (classically 6 months) for 24–36 months is recommended. Stability established at the end of follow-up is considered benign, thus the finding is relocated category 2.
4	Suspicious abnormality: finding not classic for malignancy, >2% to <95% chance of malignancy. On US and mammography, it split into: 4A (low suspiciousness of malignancy, >2% to 10%), 4B (moderate suspiciousness of malignancy, >10% to 50%), 4C (high suspiciousness of malignancy, >50% to <95%)	Intervention is essential, better to be image-guided tissue biopsy to create a pathologic diagnosis; follow-up of biopsy outcomes with radiology-pathology correlation is allocated to the examining radiologist.
5	Extremely evocative of malignancy: 95–100% likelihood of malignancy; typical findings of malignancy	Image-guided Core biopsy for tissue sample; benign result is deemed discordant, and further intervention is advised and may incorporate replicate image-guided vs surgical biopsy.
6	Biopsy-proven malignancy: verified cancer that has not finished definitive treatment	Properly utilized in patients receiving neoadjuvant therapy or in those who need additional staging; clinical managing of the malignancy is recommended.

**Table 2:** BI-RADS 5th edition mammographic findings.

Findings	Descriptors
Breast density	a. nearly entirely fatty b. scattered areas of fibroglandular density c. heterogeneously dense d. extremely dense
Mass: Shape Margin Density	A space-occupying 3D object. Round, oval, irregular Circumscribed, obscured, micro-lobulated, indistinct, spiculated High, equal density, low, fat-containing
Calcification:	
Typically benign	Skin, popcornlike vascular, large rodlike, milk of calcium, dystrophic, suture
Suspiciou morphology	Amorphous, heterogeneous, coarse, fine linear or fine-linear branching, fine pleomorphic.
Distribution	Diffuse, linear, segmental, regional, grouped.
Asymmetry	Asymmetry, global asymmetry, focal asymmetry, developing asymmetry
Associated features	Skin thickening, skin retraction, retracted nipple, trabecular thickening, axillary lymphadenopathy, calcifications
Location of lesion	Laterality, clockface and quadrant, distance from the nipple

**Table 3:** BI-RADS 5th edition ultrasound findings.

Findings	Descriptors
Tissue composition/ background echotexture	Homogenous (Fat, fibroglandular). Heterogenous.
Mass:	
Shape Margin Orientation Echo pattern Posterior features	Round, oval, irregular. Circumscribed, indistinct, angular, microlobulated, spiculated. Parallel or not parallel Anechoic, hypoechoic, hyperechoic, isoechoic, heterogeneous, complex cystic and solid. No posterior acoustic features, shadowing, enhancement, combined features.
Calcification:	Calcifications inside a mass, intraductal calcifications outside of a mass.
Associated features	Skin thickening, edema, skin retraction, vascularity (absent, vessels in rim, internal vascularity), architectural distortion, elasticity assessment (soft, intermediate, hard).
Special cases	Simple cyst, complicated cyst, clustered microcysts, foreign body counting implants, mass in skin, lymph nodes (intramammary or axillary), vascular abnormalities (arteriovenous malformation, pseudoaneurysms, or Mondor disease), postoperative collection, fat necrosis.

**Table 5:** BI-RADS 5th edition MRI findings.

Findings	Descriptors
Tissue composition	Entirely fatty breast Scattered fibroglandular tissue Heterogeneous fibroglandular tissue Marked fibroglandular tissue
Background parenchymal enhancement (BPE)	
Symmetry Level	Symmetrical/Asymmetrical Minimal/Mild/Moderate/Severe
Focus:	Yes/No
Mass:	
Shape Margin	Oval (+lobulated)/Round/Irregular Circumscribed
	Irregular/Spiculated
Patterns of internal enhancement	Homogenous Heterogenous Clumped Clustered ring
Non-mass enhancement:	
Distribution	Focal/Linear/Segmental/Regional/Multi-regional/Diffuse
Patterns of internal enhancement	Homogenous Heterogenous Clumped Clustered ring
Non-enhancing findings:	Cyst, non-enhancing mass, architectural distortion, ductal hyperintensity on precontrast T1 weighted images, postsurgical hematoma or seroma, posttreatment skin thickening, signal void from clips and foreign bodies.
Concomitant findings:	Skin retraction, skin invasion, nipple retraction, nipple invasion, pectoralis muscle invasion, chest wall invasion, inflammatory breast cancer, axillary adenopathy, architectural distortion.
Fat-containing lesions:	Normal or abnormal lymph nodes, hamartoma, fat necrosis, postoperative seroma encompassing fat.
Intra-mammary lymph nodes:	Yes/No
Skin lesions:	Yes/No
Location and depth of lesions:	
Implant findings:	Material of the implant, lumen type, contour, position, water droplets, intra- and extracapsular findings, peri-implant findings.
Kinetic signal intensity time curve assessment:	
Initial phase	Slow/Medium/Fast
Delayed phase	Persistent/Plateau/Washout

**Table 6:** Performance metrics used to assess the performance of the different AI components.

Name	Rule
Accuracy (Acc)	(TP + TN)/Total
Precision (Prec)	TP/(TP + FP)
Recall (Rec) or Sensitivity (Sens) or True Positive Rate (TPR)	TP/(TP + FN)
Specificity (Spec)	TN/(FP + TN)
F-Measure (F1-M)	(2 × Precision × Recall)/(Precision + Recall)
False Positive Rate (FPR)	FP/(FP+TN)
PR AUC	Precision-Recall Area Under Curve
Receiver operating characteristic curve (ROC)	An ROC curve plots TPR vs. FPR at different classification thresholds
Area Under the ROC Curve (AUC)	AUC measures the entire two-dimensional area underneath the entire ROC curve
Correlation	$\frac{\left(TP\times TN)-(FP\times FN\right)}{\sqrt{\left(TP+FP)(TP+FN)(TN+FP)(TN+FN\right)}}$
AUC-SD	Standard deviation of the AUC

**Table 7:** Summary of SVM-based BC detection/diagnostic methods.

Study	Method	Goal	Database	Evaluation
Adel et al., 2019 [76]	Feature extraction from both B-mode and elastography images. PCA feature reduction SVM classification	Malignant/Benign BC Classification	Private data, 82 images from 34 patients (56 malignant and 26 benign)	Acc = 94.1
Wei et al., 2019 [84]	Manual ROI segmentation Texture and morphological feature extraction SVM classification	Malignant/Benign Ultrasound BC Classification	Ultrasound dataset (472 benign, 589 malignant)	Acc = 87.3 Sens = 87.0 Spec = 87.6 Prec = 87.9
El-Azizy et al., 2019 [83]	Anisotropic filter was used for noise removal Lesion segmentation using active contour (semi-automated or fully automated) Three morphological features were extracted from the segmented lesion SVM classification	Malignant/Benign Ultrasound BC Classification	Private B-mode ultrasound dataset (216 benign, 107 malignant)	Semi-automated Acc = 96.0 Sens = 95.4 Spec = 97.2 Fully-automated Acc = 96.5 Sens = 95.3 Spec = 95.8
Rana et al., 2019 [85]	microwave signal. Different Machine learning classifiers were compared.	Automated lesion detection and classification using clinical data extracted from microwave device.	Private data, 20 healthy breasts and 23 non-healthy breasts	Acc = 55 (KNN) Acc = 53.8 (MLP) Acc = 98.9 (SVM)
Ed-daoudy and Maalmi, 2020 [82]	Feature selection using AR SVM classification	Malignant/Benign BC Classification	WBCD (357 benign, 212 malignant)	Acc = 97.0 (9 features) Acc = 96.0 (4 features) Acc = 98.0 (8 features)
Khan et al., 2021 [81]	Cytology image preprocessing Cell segmentation using GACs GLCM features were extracted from the segmented cells SVM classification	Malignant/Benign Cytology BC Classification	More than 4000 images from the pathology department Lady Reading Hospital Peshawar	Acc = 96.3
Ara et al., 2021 [77]	Less-correlated WBCD features were eliminated. Different ML classifiers were compared	Malignant/Benign BC Classification	WBCD (357 benign, 212 malignant)	Acc = 94.4 (LR) Acc = 95.8 (KNN) Acc = 95.1 (DT) Acc = 92.3 (NB) Acc = 96.5 (RF) Acc = 96.5 (SVM)
Badr et al., 2021 [79]	GWO+SVM algorithm with equilibration scaling	Malignant/Benign BC Classification BC vs. healthy classification	WBCD (357 benign, 212 malignant) HER (64 BC, 52 healthy)	Acc = 99.3 (WDBC) Acc = 93.3 (HER)
Sami et al., 2022 [86]	Transformation of the frequency domain microwave signal into time domainsignal. SVM classification.	Prediction of the breast lesion using microwave signals	Open-source datasets consisted of 1008 data examples obtained at the University of Manitoba.	Acc = 99.7 (SVM${}_{Poly}$) Acc = 98 (SVM${}_{Linear}$) Acc = 87.7 (Linear Discriminant Analysis [LDA])

**Table 8:** Summary of DT/RF-based BC detection/diagnostic methods.

Study	Method	Goal	Database	Evaluation
Singh et al., 2018 [91]	Different ML classifiers were compared	Malignant/Benign BC Classification	WBCO (456 benign, 241 malignant	Acc = 95.3 (NB) Acc = 95.8 (BLR) Acc = 97.3 (DT, J48) Acc = 98.1 (DT, CART)
Sengar et al., 2020 [93]	LR and DT classifiers were compared	Malignant/Benign BC Classification	WBCD (357 benign, 212 malignant)	Acc = 94.4 (LR) Acc = 95.1 (DT)
Allada et al., 2021 [92]	Different ML classifiers were compared	Malignant/Benign BC Classification	WBCD (357 benign, 212 malignant)	Acc = 92.3 (NB) Acc = 94.0 (LR) Acc = 95.0 (KNN) Acc = 95.1 (DT) Acc = 96.5 (SVM) Acc = 96.5 (RF)

**Table 9:** Summary of ANN/AE-based BC detection/diagnostic methods.

Study	Method	Goal	Database	Evaluation
Abbass et al., 2002 [94]	EANN based on PDE with local search	Malignant/Benign BC Classification	WBCD	Acc = 99.1
Karabatak et al., 2009 [95]	AR feature reduction ANN classification	Malignant/Benign BC Classification	WBCD	Acc = 95.6
Rouhi et al., 2015 [97]	Cellular neural network was used for tumor segmentation Features were extracted from the segmented tumors GA-based feature selection ANN classification	Malignant/Benign Mammogram BC Classification	MIAS DDSM	MAIS Acc = 90.2 Sens = 92.7 Spec = 90.5 AUC = 95.6 DDSM Acc = 96.5 Sens = 96.9 Spec = 95.9 AUC = 95.1
Jafari-Marandi et al., 2018 [96]	A SOM followed by an ANN	Malignant/Benign BC Classification	WBCD WDBC	Acc = 96.2 (WDBC) Acc = 98.2 (WBCD)
Kadam et al., 2019 [100]	Two SSAE + ensemble of softmax classifiers	Malignant/Benign BC Classification	WDBC	Acc = 98.6 Sens = 97.2 Spec = 99.4 Prec = 99.0 Rec = 97.2 F1-M = 0.98

**Table 10:** Summary of CNN-based BC detection/diagnostic methods.

Study	Method	Goal	Database	Evaluation
Arevalo et al., 2015 [101]	Cropping, augmentation, normalization CNN feature extraction SVM classification	Malignant/Benign BC Mammogram Classification	BCDR [102] (736 images from 344 patients, 426 benign, 310 malignant)	AUC = 0.86
Zhang et al., 2016 [103]	Two-layer DL architecture (PGBM+RBM)	Malignant/Benign BC Classification	227 SWE images (135 benign, 92 malignant)	Acc = 93.4 Sens = 88.6 Spec = 97.1
Huynh et al., 2016 [116]	Soft voting for two SVM outputs; one uses transfer learning Alexnet features and the other uses handcrafted features	BC Mammogram Classification	Data from University of Chicago Medical Center (607 images, 261 benign, 346 malignant)	AUC = 0.86
Araújo et al., 2017 [107]	Patch-wise classification (CNN + SVM) Majority voting for fusing the labels of the patches	Malignant/Benign BC Histopathology Classification	Online dataset [108] (249 training images, 20 testing images)	Acc = 77.8 (4 classes) Acc = 83.3 (2 classes)
Kooi et al., 2017 [109]	Integrating CNN features with handcrafted features	Detection of solid, malignant lesions from mammogram	Local dataset of around 45,000 images	Acc = 94.1
Tan et al., 2017 [110]	Preprocessing + CNN	BC Mammogram Classification	Mini-MIAS [98] (62 benign, 51 malignant, 209 normal)	Acc = 82.7 Sens = 82.7 Spec = 82.7
Agnes et al., 2019 [111]	Preprocessing + MA-CNN	BC Mammogram Classification	Mini-MIAS [98] (62 benign, 51 malignant, 209 normal)	Sens = 96.0 Spec = 96.0 F1-M = 97.0 AUC = 0.99
Ting et al., 2019 [106]	Feature-wise data augmentation and preprocessing CNN classification	BC Mammogram Classification	MIAS [98] (21 benign, 27 malignant, 183 normal)	Acc = 90.5 Sens = 89.5 Spec = 90.7
Hu et al., 2020 [118]	VGG19 was used for feature extraction and SVM for classification Three fusion levels for DCE-MRI and T2W data were investigated	BC Classification	Multiparametric data (DCE-MRI and T2W) of 927 unique breast lesions from 616 women (199 benign, 728 malignant)	AUC = 0.85 (DCE) AUC = 0.78 (T2w) AUC = 0.85 (Image fusion) AUC = 0.87 (Feature fusion) AUC = 0.86 (Classier fusion)
Hassan et al., 2020 [119]	Pre-processing using MSER [101] Pre-trained AlexNet and GoogleNet were compared for feature extraction and classification	BC Mammogram Classification	Training data CBIS-DDSM [122] INbreast [113] Test data MIAS [98] Real NCI cases	AlexNet Acc = 98.5 (MIAS) AUC = 0.99 (MIAS) Acc = 97.9 (NCI) AUC = 0.98 (NCI) GoogleNet Acc = 88.2 (MIAS) AUC = 0.95 (MIAS) Acc = 91.6 (NCI) AUC = 0.96 (NCI)
Wang et al., 2020 [123]	Pre-trained Inception-v3 models were applied for feature extraction from multi-view (transverse /coronal) US images	BC Ultrasound Classification	Private JNUH data (316 breast lesion, 181 benign, 135 malignant)	CNN A Sens = 88.6 Spec = 87.6 AUC = 0.95 CNN B Sens = 86.5 Spec = 84.8 AUC = 0.93
Wang et al., 2021 [104]	Fusion of CNN features with capsule network features Classification using a modified capsule network	Malignant/Benign BC Histopathology Classification	BreaKHis [105] (135 benign, 92 malignant)	Acc = 95.6
Muduli et al., 2021 [112]	Preprocessing + CNN	BC Classification	Mammogram MAIS, 326 images DDSM, 1500 images INbreast, 410 images Ultrasound BUS-1780 images BUS-2250 images	Acc = 96.5 (MIAS) Acc = 90.7 (DDSM) Acc = 90.7 (INbreast) Acc = 90.7 (BUS-1) Acc = 90.7 (BUS-2)
Hekal et al., 2021 [125]	Otsu segmentation of suspected regions, AlexNet/ResNet for feature extraction, and SVM for classification	Classification of benign and malignant mammogram structures	CBIS-DDSM [122] detest of 3549 mammogram images (1852 benign, 1697 malignant)	Acc = 91.0 (AlexNet) Acc = 84.0 (ResNet-50)
Haq et al., 2022 [115]	Unsharp Image enhancement ROI extraction via canny detector Ensemble of a CNN with three parallel classifiers with majority voting	BC Mammogram Classification	MIAS [98] BCDR [129]	MIAS Acc = 99.4 Sens = 99.5 Spec = 99.4 BCDR Acc = 98.5 Sens = 98.8 Spec = 98.4
Moreau et al., 2022 [128]	U-Net model Evaluate the treament response using four different biomarkers: standardized uptake value peak(SUL${}_{peak}$), total lesion glycolysis (TLG), PET bone index (PBI) and PET liver index (PLI).	Automatic Segmentation of Metastatic Breast Cancer for Treatment Response Assessment.	Images were acquired at two sites (A-ICO, N-ICO).	SUL${}_{peak}$ Sens = 87 Spec = 87 TLG Sens = 73 Spec = 81 PBI Sens = 69 Spec = 69
Hekal et al., 2023 [127]	Otsu segmentation of suspected regions followed by ensemble DL	Classification of benign and malignant mammogram structures	CBIS-DDSM [122] detest of 3549 mammogram images (1852 benign, 1697 malignant)	Acc = 94.0 (Benign vs. Malignant) Acc = 95.0 (Benign vs. Malignant Mass)

**Table 11:** The most frequent modalities, features, and AI/ML components used for breast cancer (BC) detection and diagnosis.

Modalities/Database	Features	AL/ML Components
Mammogram Ultrasound Elastography Histopathology MRI Multi-parametric data Cell characteristics Patient Record	Statistical Morphological Appearance Texture Cell tissue features Health records Deep learning	LR KNN NB SVM DT/RF ANN ANN AE CNN

**Table 12:** Imaging-based breast biomarkers. ER: estrogen receptor PR: progesterone receptor, HER2: human epidermal growth factor receptor 2.

Type of Biomarker	Examples
Diagnostic	ER, PR, HER2 and BI-RADS descriptors
Pharmacodynamic	Standardized uptake value (SUV) at 18-FDG PET-CT and 68Ga-FAPI SUVmax
Predictive	ER, PR, BRCA gene, increased mammographic breast density
Prognostic	Tumor stage, grade, tumor receptor status, and SUV at 18-FDG PET-CT

## Data Availability

Not applicable.

## Abbreviations

The following abbreviations are used in this manuscript:

AI	Artificial Intelligence
ACR	American College of Radiology
AdaBoost	Adaptive Boosting
ADC	Apparent diffusion coefficient
AE	Autoencoder
ALN	Axillary lymph node
ANN	Artificial neural networks
AR	Association rules
AUC	Area under the ROC curve
BI-RADS	Breast Imaging Reporting and Data System
BC	Breast cancer
BCDR	Breast cancer digital repository
BLR	Bayesian logistic regression
BreaKHis	Breast cancer histopathological database
CAE	Contractive autoencoder
CAD	Computer aided diagnosis
CART	Classification and Regression Trees
CEM	Contrast-enhanced mammography
CEUS	Contrast-enhanced ultrasound
CNN	Convolutional neural network
DAE	Denoising autoencoder
DCE-MRI	Dynamic contrast-enhanced magnetic resonance imaging
DDSM	Digital database for screening mammography
DL	Deep learning
DT	Decision Tree
DTI-MRI	Diffusion tensor magnetic resonance imaging
DSS	Disease-specific survival
DW-MRI	Diffusion weighted magnetic resonance imaging
EANN	Evolutionary artificial neural network
EP	Evolutionary programming
ER	Estrogen receptor
FA	Fractional anisotropy
FN	False negative
FP	False positive
GA	Genetic algorithm
GAC	Geometric active contour
GBoost	Gradient Boosting
GLCM	Gray level co-occurrence matrix
GLMNet	Generalized linear regression with elastic net
GNB	Gaussian Naïve Bayes
GWO	Grey wolf optimization
HER	Electronic health record
HER2	Human epidermal growth factor receptor 2
HOMA	homeostatic model assessment
ID3	Iterative Dichotomiser 3
JNUH	Jeonbuk national university hospital
KNN	K-Nearest Neighbor
MA-CNN	Multiscale all CNN
MBI	Molecular breast imaging
LDA	Linear discriminant analysis
LR	Logistic Regression
MCP-1	Monocyte chemoattractant protein-1
MIAS	Mammographic image analysis society database
ML	Machine learning
MLP	Multi-layer perceptron
MRI	Magnetic Resonance Imaging
MRMR	Maximum relevance minimum redundancy
MSER	Maximally stable extremal regions
NAC	Neoadjuvant chemotherapy
NCI	National Cancer Institute
NB	Naïve Bayes
PDE	Pareto-differential evolution algorithm
PEM	Positron emission mammography.
RF	Random forest
RFE-RF	Recursive feature elimination random forest
RFS	Recurrence-free survival
pCR	Pathological complete response
PGBM	Point-wise gated Boltzmann machine
PR	Progesterone receptor
QDA	Quadratic Discriminant Analysis
ACR	American College of Radiology
RBM	Restricted Boltzmann machine
RCB	Residual cancer burden
ROC	Receiver operating characteristic
ROI	Region of interest
SAE	Sparse autoencoder
SGD	Stochastic gradient descent
SOM	Self organization map
SSAE	Stacked sparse autoencoder
SWE	Shear-wave elastography
TN	True negative
TP	True positive
UCI	University of California Irvine
US	Ultrasonography
VAE	Variational autoencoder
WBCD	Wisconsin breast cancer database
WBCO	Wisconsin breast cancer original database
WSAW	Weighted simple additive weighting
WSE	Wrapper subset evaluator
XGBoost	Extreme gradient boosting
